# Supplementary figures and images for: Modeling chronic wasting disease transmission risk in mule deer related to habitat characteristics
Source: PLoS One. 2026 Apr 29;21(4):e0346077. doi: 10.1371/journal.pone.0346077 (PMC13127966; doi:10.1371/journal.pone.0346077)

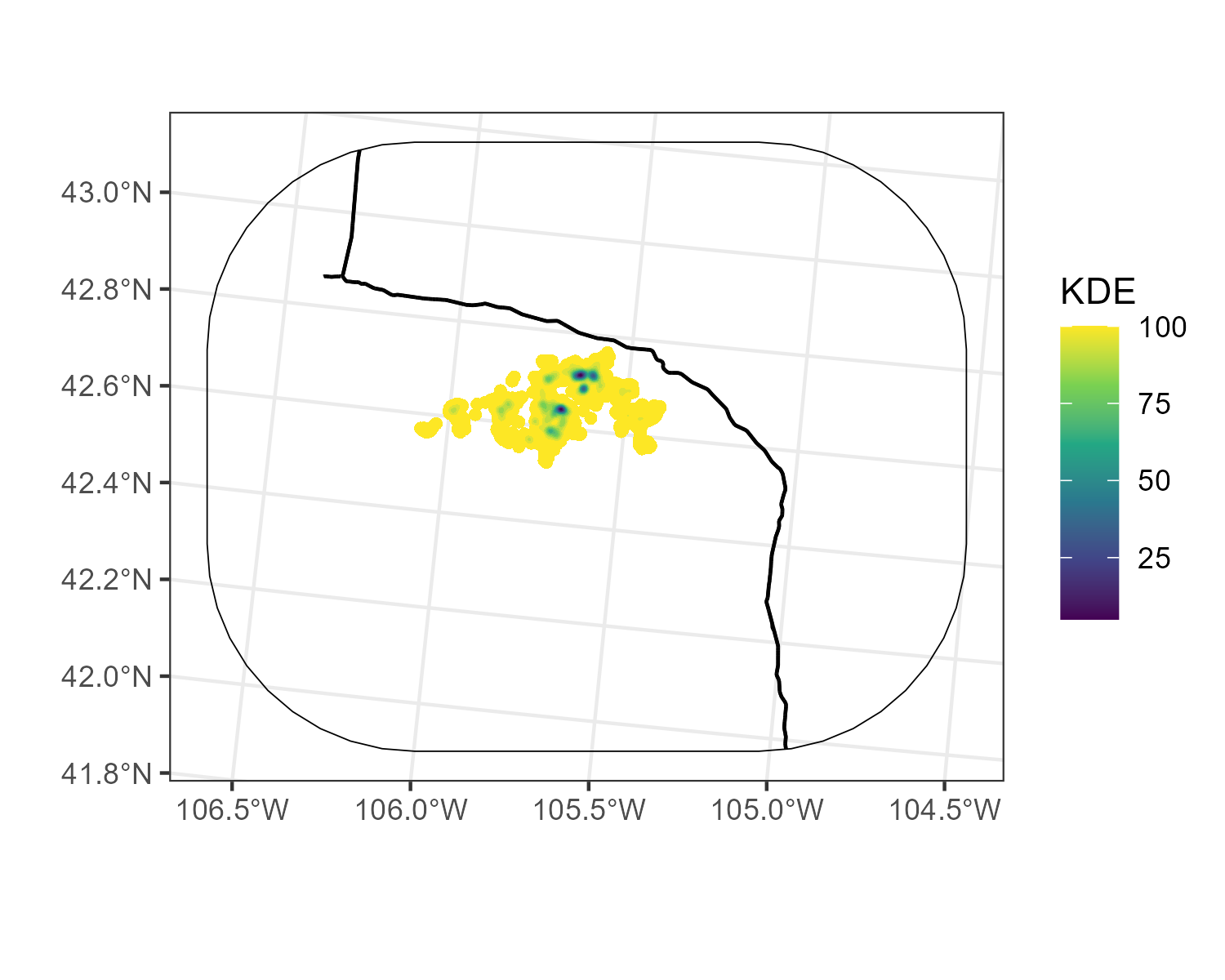

Supplement: S1 Fig — The black solid line bisecting the study area is Interstate 25. (PNG) [file pone.0346077.s001.png]

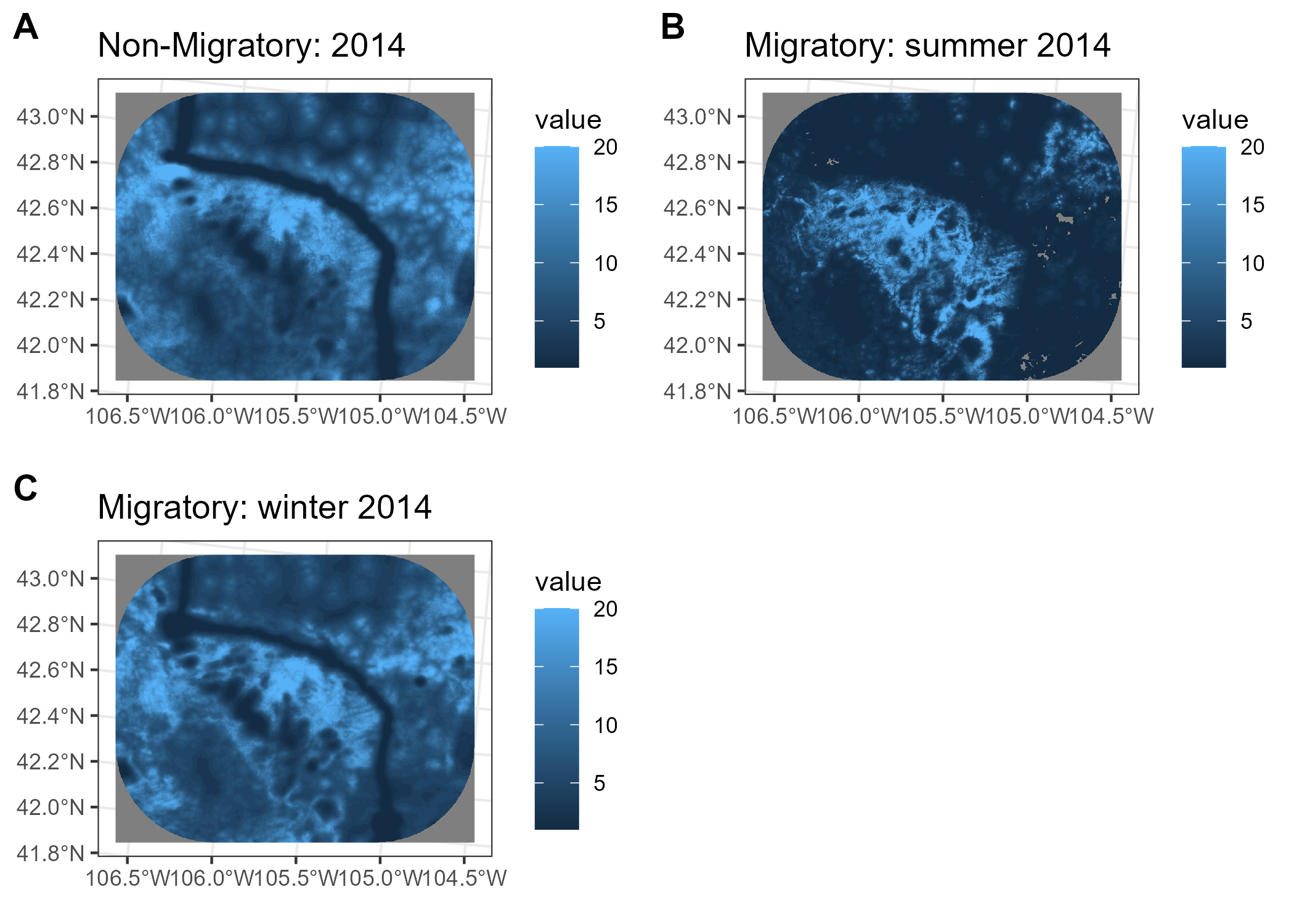

Supplement: S5 Fig — A value of 20 indicates the most suitable habitat. Resource selection functions (RSFs) were fit separately for A) nonmigratory deer, B) migratory deer on summer range, and C) migratory deer on winter range. Predictions depend on year-specific spatial covariates; maps are shown based on data from 2014 (refer to S1 File for descriptions and citations for data used in modeling). (PNG) [file pone.0346077.s005.png]

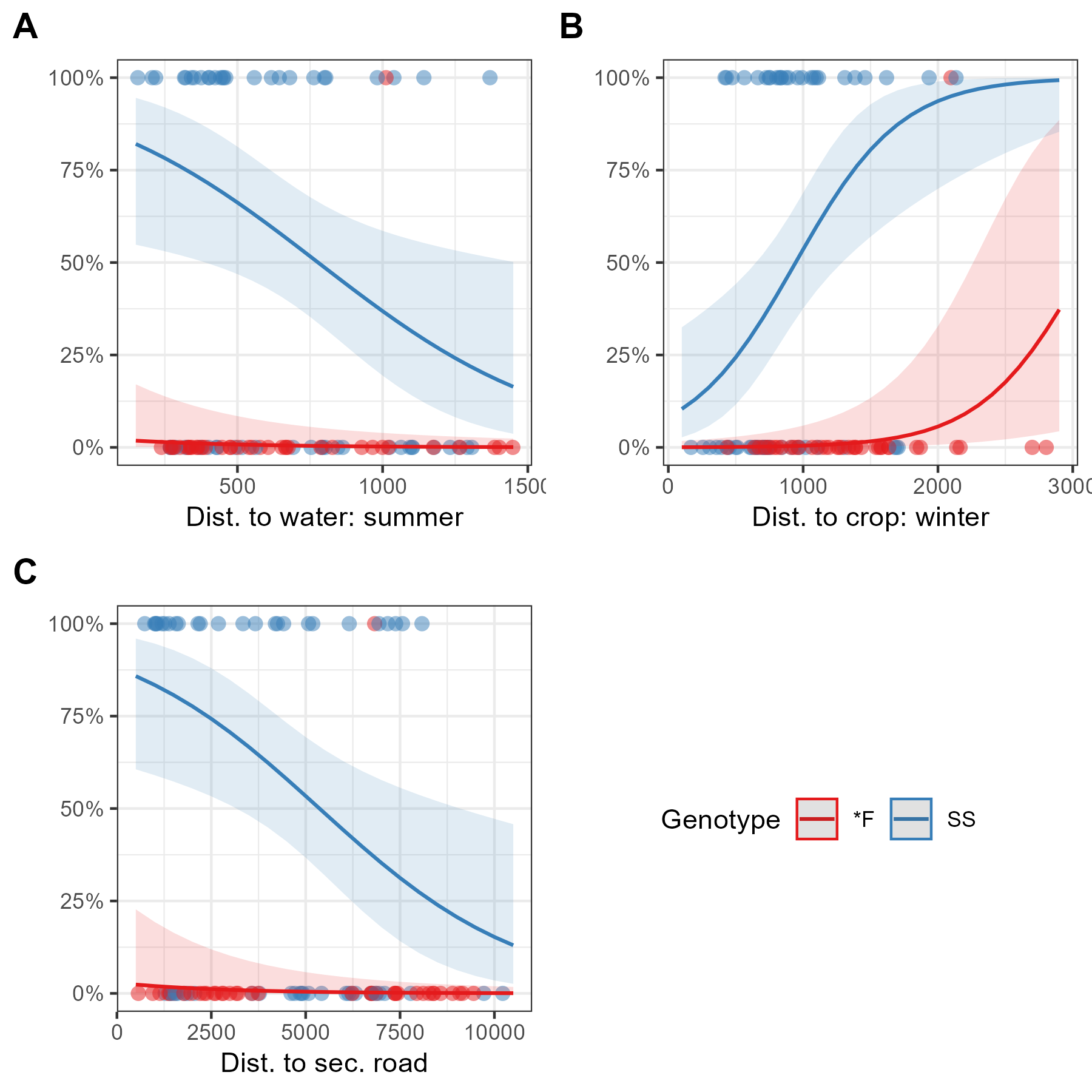

Supplement: S6 Fig — Covariates include A) distance to perennial water source during summer, B) distance to cropland during winter, and C) distance to secondary road. Color indicates differences in effect by genotype (*F and SS). (TIF) [file pone.0346077.s006.tif]

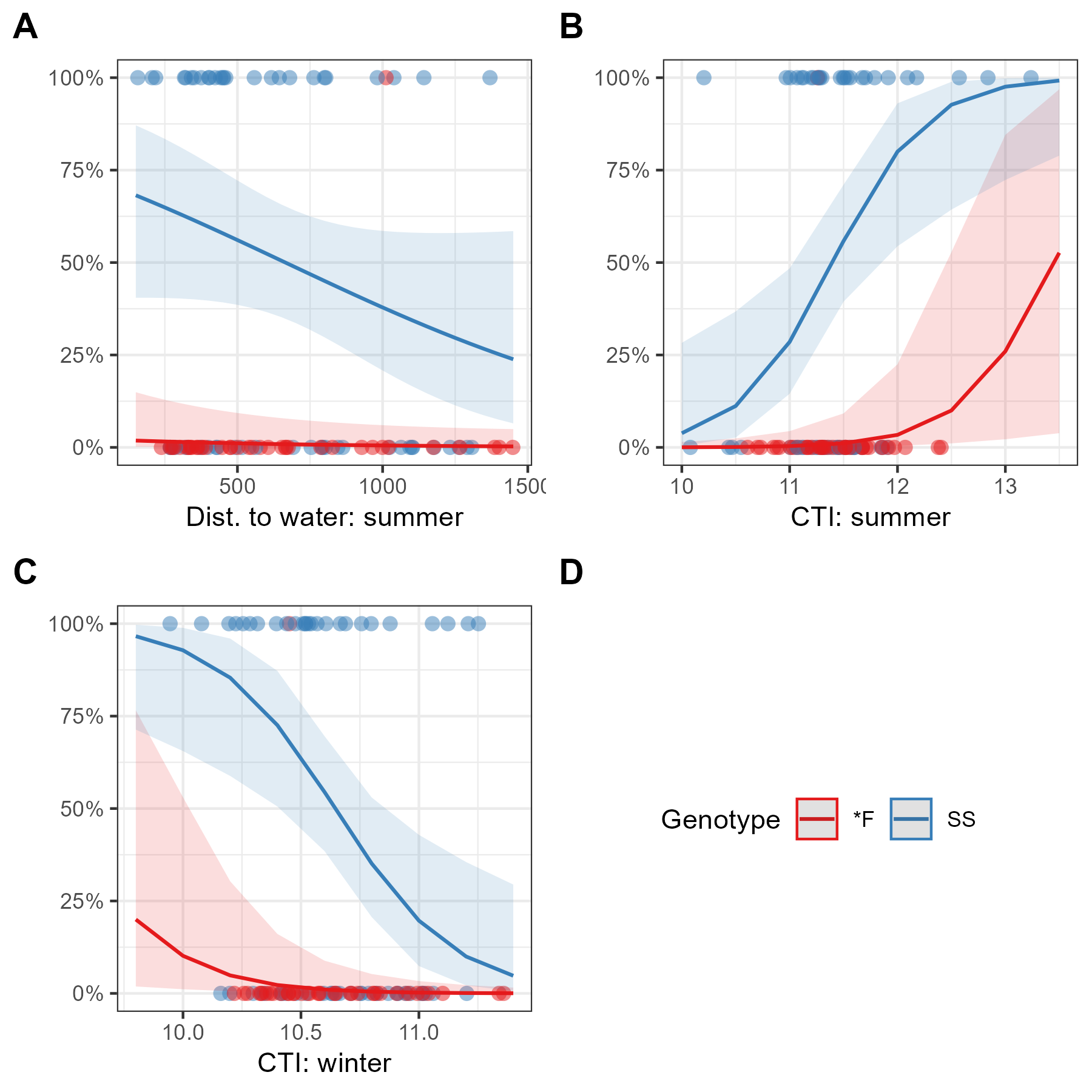

Supplement: S7 Fig — Covariates include A) distance to perennial water source during summer, B) compound topographic index (CTI) during summer, and C) CTI during winter. Color indicates differences in effect by genotype (*F and SS). (TIF) [file pone.0346077.s007.tif]

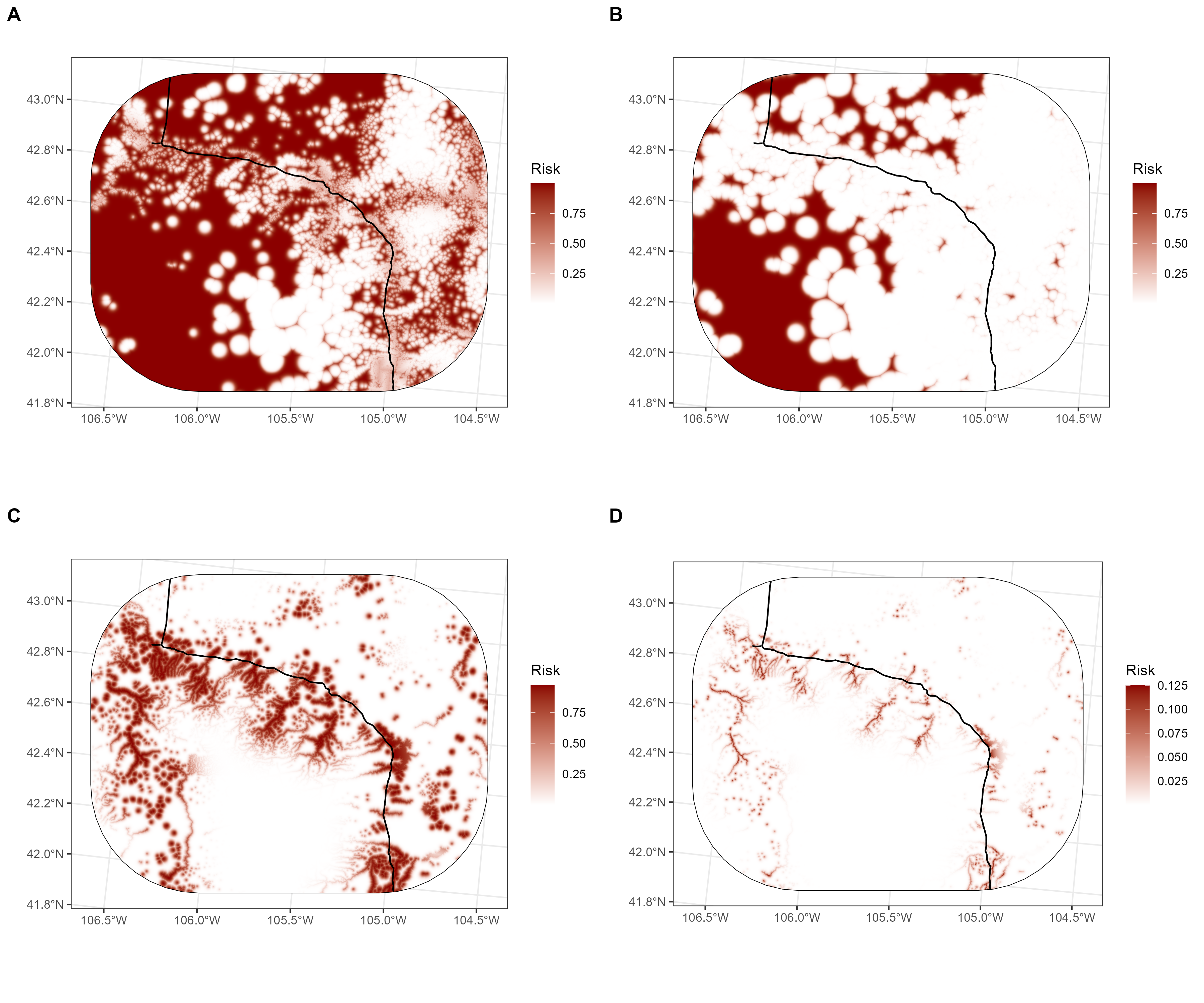

Supplement: S8 Fig — The black solid line bisecting the study area is Interstate 25. Maps are specific to genotype and season: A) genotype SS, winter, B) genotype *F, winter, C) genotype SS, summer, D) genotype *F, summer. (PNG) [file pone.0346077.s008.png]

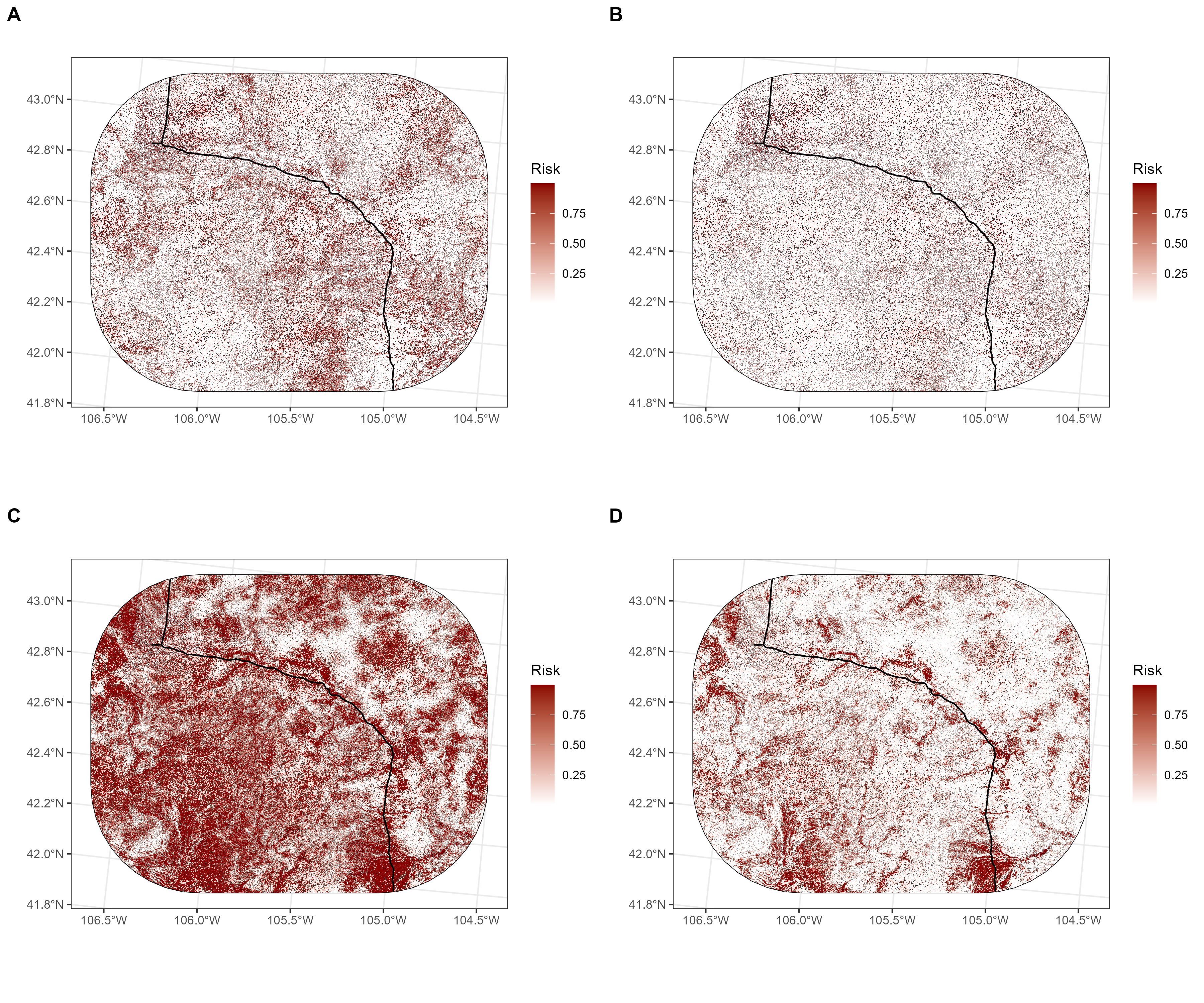

Supplement: S9 Fig — The black solid line bisecting the study area is Interstate 25. Maps are specific to genotype and season: A) genotype SS, winter, B) genotype *F, winter, C) genotype SS, summer, D) genotype *F, summer. (PNG) [file pone.0346077.s009.png]

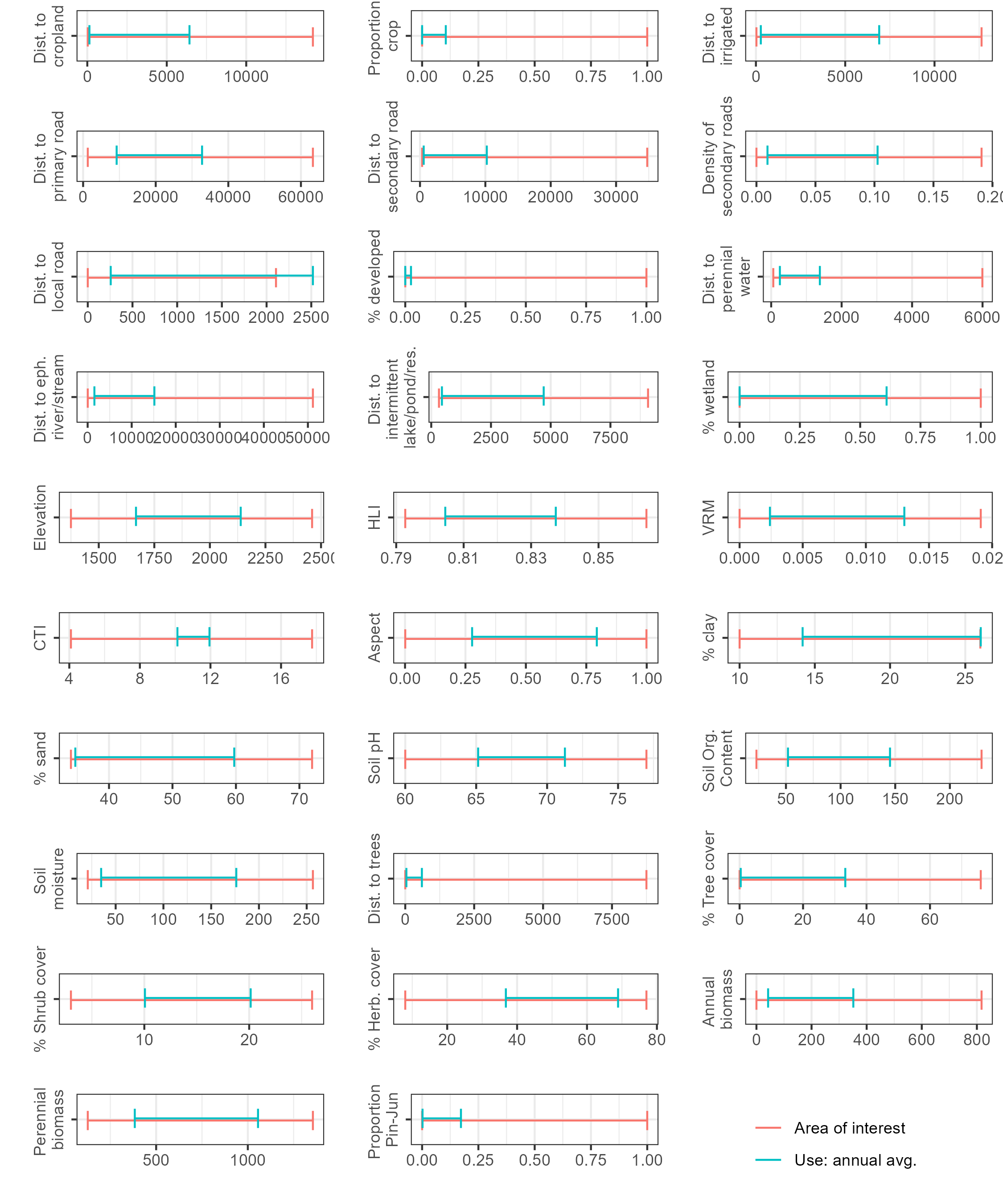

Supplement: S10 Fig — (TIF) [file pone.0346077.s010.tif]
