# Supplementary material for: Modeling chronic wasting disease transmission risk in mule deer related to habitat characteristics
Source: PLoS One. 2026 Apr 29;21(4):e0346077. doi: 10.1371/journal.pone.0346077 (PMC13127966; doi:10.1371/journal.pone.0346077)
Supplement: S1 Table — Risk models used data from 59 female mule deer from the South Converse Mule Deer Herd. Each deer was equipped with a GPS collar which recorded locations every 6 or 8 hours for 1–4 years. (PDF) [file pone.0346077.s011.pdf]

**Length of data record**	**Number of deer**	**Deer ID**
**1 year**	38	2, 3, 12, 15, 16, 21, 24, 27, 31, 33, 35, 38, 43, 60, 61, 64, 66, 67, 68, 69, 71, 79, 83, 84, 88, 91, 96, 99, 104, 109, 111, 113, 114, 115, 119, 121, 124, 130
**2 years**	10	13, 34, 51, 56, 76, 77, 78, 82, 89, 100
**3 years**	8	1, 18, 23, 30, 42, 45, 50, 72
**4 years**	3	4, 7, 17
**Total**	**59**	
