# Supplementary material for: Modeling chronic wasting disease transmission risk in mule deer related to habitat characteristics
Source: PLoS One. 2026 Apr 29;21(4):e0346077. doi: 10.1371/journal.pone.0346077 (PMC13127966; doi:10.1371/journal.pone.0346077)
Supplement: S2 Table — Location data used in constructing the model was collected from 2010–2013 as part of a chronic wasting disease study. “Form” indicates the structure of the covariate. “Proportion” indicates the amount of a categorical variable within the specified moving window, refer to “scale;” “Mean” refers to the average value of a continuous covariate over the specified moving window, refer to “scale;” ”Euclidian” is the straight-line distance to the nearest feature of the covariate of interest; and “Decay” is a distance metric calculated using a decay function where the effect diminishes as the distance from the feature increases. The rate of decay is specified under “scale.” “β” and “SE” indicate each parameter estimate and standard error respectively. Note that positive coefficients associated with Euclidean distance measures indicate that predicted mule deer use is farther from the feature while negative coefficients indicate that predicted use is closer to the feature. The opposite relationship is true for decay distance measures. (PDF) [file pone.0346077.s012.pdf]

| Variable | | Form | Scale | β | SE | P value |
|---|---|---|---|---|---|---|
| Agriculture | | | | | | |
| | Proportion of area designated as cropland - linear | Proportion, quadratic | 2,548m | -0.2405 | 0.0352 | 0.0000 |
| | Proportion of area designated as cropland - quadratic | | | -0.0898 | 0.0679 | 0.1454 |
| | Distance to cropland | Euclidean | Pixel value | -1.4306 | 0.0486 | 0.0000 |
| | Distance to irrigated land | Euclidean | Pixel value | -2.8685 | 0.0636 | 0.0000 |
| Roads | | | | | | |
| | Distance to local or 4WD road | Decay | 1,000m | -0.2443 | 0.0103 | 0.0000 |
| | Distance to primary road | Decay | 1,515m | -3.7614 | 0.1660 | 0.0000 |
| | Distance to secondary road | Euclidean | Pixel value | -1.4078 | 0.0538 | 0.0000 |
| | Density of secondary roads - linear | Linear density, quadratic | 2,548m | 0.8902 | 0.0618 | 0.0000 |
| | Density of secondary roads - quadratic | | | -0.7345 | 0.0676 | 0.0000 |
| Terrain | | | | | | |
| | Aspect | N/A | Pixel value | 0.2753 | 0.0075 | 0.0000 |
| | Compound topographic index (CTI) | Mean | 250m | -0.0372 | 0.0139 | 0.0075 |
| | Heat load index (HLI) | Mean | 50m | -0.6111 | 0.0127 | 0.0000 |
| | Vector ruggedness (VRM) | Mean | 100m | -0.1277 | 0.0110 | 0.0000 |
| Vegetation | | | | | | |
| | Annual vegetation biomass | Mean | 2,548m | 0.0071 | 0.0097 | 0.4668 |
| | Perennial vegetation biomass | Mean | 2,548m | -0.1267 | 0.0149 | 0.0000 |
| | Percent shrub cover (RCMAP)[1] – linear | Mean, quadratic | 2,548m | 11.2216 | 0.1885 | 0.0000 |
| | Percent shrub cover (RCMAP) – quadratic | | | -10.6752 | 0.1835 | 0.0000 |
| | Percent tree cover (RCMAP) – linear | Mean, quadratic | 2,548m | 2.2096 | 0.1364 | 0.0000 |
| | Percent tree cover (RCMAP) – quadratic | | | -8.9320 | 0.3966 | 0.0000 |
| | Proportion of area designated as early growth conifer/pinyon-juniper (RCMAP) | Proportion | 1,515m | -0.5756 | 0.0202 | 0.0000 |
| | Distance to trees (>1% cover) (NLCD)[2] | Euclidean | Pixel value | -1.0368 | 0.0199 | 0.0000 |
| Water | | | | | | |
| | Distance to ephemeral stream/river | Decay | 500m | -2.3297 | 0.0965 | 0.0000 |
| | Distance to intermittent lake/pond/reservoir | Decay | 2,548m | 0.2011 | 0.0116 | 0.0000 |
| | Distance to perennial stream/river | Decay | 1,000m | -0.3832 | 0.0118 | 0.0000 |
| | Distance to perennial lake/pond/reservoir | Decay | 1,000m | 0.3779 | 0.0082 | 0.0000 |
| | Distance to spring/seep | Euclidean | Pixel value | -0.0382 | 0.0107 | 0.0004 |

[1]U.S. Geological Survey, Rigge, M., Bunde, B., Postma, K., and Shi, H. 2024. Rangeland Condition Monitoring Assessment and Projection (RCMAP) Herbaceous fractional component time-series across Western North America from 1985-2023. U.S. Geological Survey data release, https://doi.org/10.5066/P9SJXUI1
[2]Dewitz, J. 2023. National Land Cover Database (NLCD) 2021 Products. U.S. Geological Survey data release, https://doi.org/10.5066/P9JZ7AO3
