# Supplementary material for: Modeling chronic wasting disease transmission risk in mule deer related to habitat characteristics
Source: PLoS One. 2026 Apr 29;21(4):e0346077. doi: 10.1371/journal.pone.0346077 (PMC13127966; doi:10.1371/journal.pone.0346077)
Supplement: S4 Table — Location data used in constructing the model was collected from 2010–2013 as part of a chronic wasting disease study. “β” and “SE” indicate each parameter estimate and standard error respectively. Note that positive coefficients associated with Euclidean distance measures indicate that predicted mule deer use is farther from the feature while negative coefficients indicate that predicted use is closer to the feature. The opposite relationship is true for decay distance measures. (DF) [file pone.0346077.s014.pdf]

Variable		Form	Scale	β	SE	P value
Agriculture						
	Proportion of area designated as cropland - linear	Proportion, quadratic	2,548m	1.1500	0.0599	0.0000
	Proportion of area designated as cropland - quadratic			-2.0776	0.1376	0.0000
	Distance to cropland	Euclidean	Pixel value	-1.0580	0.0647	0.0000
	Distance to irrigated land	Euclidean	Pixel value	-1.7587	0.0764	0.0000
Roads						
	Distance to local or 4WD road	Decay	500m	-0.1141	0.0150	0.0000
	Distance to primary road	Decay	1,515m	-6.1321	0.3073	0.0000
	Distance to secondary road	Euclidean	Pixel value	-2.2146	0.0946	0.0000
	Density of secondary roads - linear	Linear density, quadratic	2,548m	3.9267	0.1096	0.0000
	Density of secondary roads - quadratic			-4.2522	0.1313	0.0000
Terrain						
	Aspect	Mean	1,000m	0.9496	0.0130	0.0000
	Compound topographic index (CTI)	Mean	1,000m	0.2606	0.0267	0.0000
	Heat load index (HLI)	Mean	50m	-0.2584	0.0145	0.0000
	Vector ruggedness (VRM)	Mean	500m	0.1400	0.0193	0.0000
Vegetation						
	Annual vegetation biomass	Mean	2,548m	0.1322	0.0133	0.0000
	Perennial vegetation biomass	Mean	1,515m	-0.3158	0.0191	0.0000
	Percent shrub cover (RCMAP)[1] - linear	Mean, quadratic	2,548m	16.5468	0.3086	0.0000
	Percent shrub cover (RCMAP) - quadratic			-13.4851	0.2905	0.0000
	Percent tree cover (RCMAP) - linear	Mean, quadratic	2,548m	-1.6021	0.2904	0.0000
	Percent tree cover (RCMAP) - quadratic			-15.4598	1.0620	0.0000
	Proportion of area designated as early growth conifer/pinyon-juniper (RCMAP)	Proportion	1,515m	-0.3999	0.0276	0.0000
	Distance to trees (>1% cover) (NLCD)[2]	Euclidean	Pixel value	-1.0316	0.0300	0.0000
Water						
	Distance to perennial stream/river	Decay	1,000m	-0.3546	0.0183	0.0000
	Distance to perennial lake/pond/reservoir	Decay	2,548m	0.0629	0.0149	0.0000
	Distance to spring/seep	Decay	500m	-0.3863	0.0180	0.0000

[1]U.S. Geological Survey, Rigge, M., Bunde, B., Postma, K., and Shi, H. 2024. Rangeland Condition Monitoring Assessment and Projection (RCMAP) Herbaceous fractional component time-series across Western North America from 1985-2023. U.S. Geological Survey data release, https://doi.org/10.5066/P9SJXUI1

[2]Dewitz, J. 2023. National Land Cover Database (NLCD) 2021 Products. U.S. Geological Survey data release, https://doi.org/10.5066/P9JZ7AO3
