# Supplementary material for: Modeling chronic wasting disease transmission risk in mule deer related to habitat characteristics
Source: PLoS One. 2026 Apr 29;21(4):e0346077. doi: 10.1371/journal.pone.0346077 (PMC13127966; doi:10.1371/journal.pone.0346077)
Supplement: S5 Table — K indicates the number of parameters in each model, and AICc refers to Akaike’s Information Criterion for small sample sizes. (PDF) [file pone.0346077.s015.pdf]

**Model name**	**K**	**AIC_c**
genotype	2	88.27
genotype + year + age	6	88.53
genotype + year	5	89.05
genotype + age	3	89.77
genotype + mig/nonmig	3	90.24
genotype + year + mig/nonmig	6	91.32
migratory/nonmig	2	114.48
~ 1	1	114.78
age	2	116.63
observation year	4	120.77
