# Supplementary material for: Modeling chronic wasting disease transmission risk in mule deer related to habitat characteristics
Source: PLoS One. 2026 Apr 29;21(4):e0346077. doi: 10.1371/journal.pone.0346077 (PMC13127966; doi:10.1371/journal.pone.0346077)
Supplement: S6 Table — (PDF) [file pone.0346077.s016.pdf]

	**Estimate**	**Std. Error**	**95% Confidence interval**	
(Intercept)	-3.664	1.013	-6.540	-2.138
genotype_categorySS	3.589	1.049	1.951	6.500
