# Supplementary material for: Modeling chronic wasting disease transmission risk in mule deer related to habitat characteristics
Source: PLoS One. 2026 Apr 29;21(4):e0346077. doi: 10.1371/journal.pone.0346077 (PMC13127966; doi:10.1371/journal.pone.0346077)
Supplement: S7 Table — (PDF) [file pone.0346077.s017.pdf]

	Estimate	Std. Error	95% Confidence interval	
(Intercept)	-3.807	1.028	-6.700	-2.246
genotype_categorySS	3.791	1.072	2.101	6.728
scale(mean_kde)	-0.365	0.278	-0.932	0.172
