# Supplementary material for: Modeling chronic wasting disease transmission risk in mule deer related to habitat characteristics
Source: PLoS One. 2026 Apr 29;21(4):e0346077. doi: 10.1371/journal.pone.0346077 (PMC13127966; doi:10.1371/journal.pone.0346077)
Supplement: S8 Table — PRNP genotype was included in all models. A covariate estimate of “NA” indicates the covariate was not included in the specified model. RCMAP refers to data from the Rangeland Condition Monitoring Assessment and Projection project (Rigge et al. 2024). (PDF) [file pone.0346077.s018.pdf]

Model rank	Intercept	Genotype	Biomass: annuals	Biomass: perennials - winter	Density secondary road - winter	Distance to crop - winter	Distance to irrigated - summer	Distance to perennial water - summer
1	-5.213	+	NA	NA	NA	1.351	NA	-0.8398
2	-5.242	+	NA	NA	NA	1.228	NA	-0.9098
3	-5.168	+	NA	-0.4322	NA	1.086	NA	-0.7409
4	-5.316	+	NA	NA	NA	1.312	NA	-0.8366
5	-5.297	+	NA	NA	0.2319	1.422	NA	-0.7338
6	-5.242	+	NA	NA	NA	1.321	NA	-0.8691
7	-5.376	+	NA	NA	NA	1.307	NA	-1.0090
8	-5.226	+	NA	NA	NA	1.358	NA	-0.9270
9	-5.306	+	NA	NA	NA	1.324	NA	-0.9300
10	-5.311	+	NA	NA	NA	1.358	NA	-0.8803
11	-5.458	+	NA	NA	NA	1.183	NA	-0.9217
12	-5.304	+	NA	NA	NA	1.375	NA	-0.8940
13	-5.193	+	NA	NA	NA	1.353	NA	-0.8275
14	-5.202	+	NA	NA	NA	1.348	NA	-0.8333
15	-5.207	+	NA	NA	NA	1.360	-0.03079	-0.8528

Continued

Model rank	Distance to perennial water - winter	Distance to primary road	Distance to local road - summer	Distance to secondary road	RSF	% developed	% wetland - summer	Proportion cropland - winter	Proportion early PJ - summer
1	NA	NA	NA	-1.0150	NA	NA	NA	NA	NA
2	NA	NA	NA	-1.1166	NA	NA	NA	-0.3558	NA
3	NA	NA	NA	-0.8984	NA	NA	NA	NA	NA
4	NA	NA	NA	-0.9178	NA	NA	0.3649	NA	NA
5	NA	NA	NA	-0.9220	NA	NA	NA	NA	NA
6	NA	NA	NA	-0.9834	NA	NA	NA	NA	NA
7	0.2933	NA	NA	-1.0721	NA	NA	NA	NA	NA
8	NA	NA	0.1731	-1.0328	NA	NA	NA	NA	NA
9	NA	NA	NA	-0.9960	NA	NA	NA	NA	NA
10	NA	NA	NA	-0.9449	NA	NA	NA	NA	-0.2328
11	NA	NA	NA	-1.0176	NA	NA	0.4451	-0.3945	NA
12	NA	NA	NA	-0.9427	NA	NA	NA	NA	NA
13	NA	NA	NA	-0.9667	0.111	NA	NA	NA	NA
14	NA	NA	NA	-0.9859	NA	0.06997	NA	NA	NA
15	NA	NA	NA	-1.0035	NA	NA	NA	NA	NA

Continued

Model rank	Proportion early PJ - winter	RCMAP % shrub - summer	RCMAP % tree - winter	df	logLik	AICc	Delta AICc	weight
1	NA	NA	NA	5	-32.81	76.30	0.0000	0.0021200
2	NA	NA	NA	6	-32.04	77.05	0.7497	0.0014572
3	NA	NA	NA	6	-32.45	77.86	1.5593	0.0009721
4	NA	NA	NA	6	-32.46	77.88	1.5791	0.0009626
5	NA	NA	NA	6	-32.58	78.12	1.8154	0.0008553
6	NA	NA	-0.1876	6	-32.60	78.16	1.8626	0.0008353
7	NA	NA	NA	6	-32.60	78.17	1.8677	0.0008332
8	NA	NA	NA	6	-32.66	78.29	1.9901	0.0007838
9	-0.1693	NA	NA	6	-32.69	78.35	2.0481	0.0007614
10	NA	NA	NA	6	-32.71	78.39	2.0891	0.0007459
11	NA	NA	NA	7	-31.56	78.42	2.1131	0.0007370
12	NA	-0.1387	NA	6	-32.74	78.45	2.1529	0.0007225
13	NA	NA	NA	6	-32.76	78.49	2.1874	0.0007101
14	NA	NA	NA	6	-32.80	78.56	2.2545	0.0006867
15	NA	NA	NA	6	-32.81	78.58	2.2786	0.0006785
