# Supplementary material for: Modeling chronic wasting disease transmission risk in mule deer related to habitat characteristics
Source: PLoS One. 2026 Apr 29;21(4):e0346077. doi: 10.1371/journal.pone.0346077 (PMC13127966; doi:10.1371/journal.pone.0346077)
Supplement: S9 Table — Top model included genotype, distance to cropland during winter, distance to perennial water source during summer, and distance to secondary road. Continuous covariates were standardized prior to model fit. (PDF) [file pone.0346077.s019.pdf]

	Estimate	Std. Error	95% Confidence interval	
(Intercept)	-5.213	1.298	-8.529	-3.175
genotype_categorySS	5.513	1.414	3.236	9.023
scale(mean_dist_cropwin)	1.351	0.429	0.565	2.274
scale(mean_dist_pwatersum)	-0.840	0.359	-1.607	-0.181
scale(mean_dist_road_sec)	-1.015	0.389	-1.859	-0.307
