# Supplementary material for: Modeling chronic wasting disease transmission risk in mule deer related to habitat characteristics
Source: PLoS One. 2026 Apr 29;21(4):e0346077. doi: 10.1371/journal.pone.0346077 (PMC13127966; doi:10.1371/journal.pone.0346077)
Supplement: S10 Table — PRNP genotype was included in all models. A covariate estimate of “NA” indicates the covariate was not included in the specified model. CTI = Compound Topographic Index; HLI = Heat Load Index; SOC = Surface Organic Carbon; VRM = Vector Ruggedness Measure. (PDF) [file pone.0346077.s020.pdf]

Model rank	Intercept	Genotype	Aspect - summer	% clay - winter	CTI - summer	CTI - winter	Elevation - winter	HLI - winter
1	-4.721	+	NA	NA	1.155	-1.196	NA	NA
2	-4.523	+	NA	-0.4685	1.156	-1.385	NA	NA
3	-4.391	+	NA	NA	1.181	-1.123	NA	NA
4	-4.426	+	-0.3666	NA	1.145	-1.150	NA	NA
5	-4.678	+	NA	-0.7822	1.179	-1.427	NA	NA
6	-4.580	+	NA	-0.6090	1.144	-1.310	NA	NA
7	-4.694	+	NA	-0.3414	1.132	-1.361	NA	NA
8	-4.524	+	-0.2810	-0.4010	1.126	-1.358	NA	NA
9	-4.811	+	NA	NA	1.012	-1.386	NA	NA
10	-4.387	+	NA	NA	1.194	-1.219	NA	NA
11	-4.577	+	NA	-0.4930	1.062	-1.364	NA	NA
12	-4.535	+	NA	-0.5433	1.134	-1.328	NA	NA
13	-4.798	+	NA	NA	1.070	-1.153	NA	NA
14	-4.927	+	NA	NA	1.117	-1.212	NA	NA
15	-4.652	+	NA	NA	1.165	-1.235	NA	NA

Continued:

Model rank	Distance to perennial water - summer	Distance to perennial water - winter	% wetland - summer	Soil pH - winter	% sand - summer	% sand - winter
1	-0.5128	NA	NA	NA	NA	NA
2	NA	NA	NA	NA	NA	NA
3	NA	NA	NA	NA	NA	NA
4	NA	NA	NA	NA	NA	NA
5	NA	NA	NA	NA	NA	-0.4697
6	NA	NA	NA	NA	NA	NA
7	-0.3882	NA	NA	NA	NA	NA
8	NA	NA	NA	NA	NA	NA
9	-0.5474	NA	NA	NA	NA	NA
10	NA	NA	NA	-0.2995	NA	NA
11	NA	NA	0.2986	NA	NA	NA
12	NA	NA	NA	NA	NA	NA
13	-0.5163	NA	0.2790	NA	NA	NA
14	-0.6721	0.2808	NA	NA	NA	NA
15	-0.4465	NA	NA	-0.1627	NA	NA

Continued:

**Model rank**	**SOC**	**SOC - winter**	**VRM**	**df**	**logLik**	**AICc**	**delta**	**weight**
1	NA	NA	NA	5	-33.42	77.53	0.0000	0.006237
2	NA	NA	NA	5	-33.48	77.64	0.1071	0.005912
3	NA	NA	NA	4	-34.81	78.07	0.5455	0.004748
4	NA	NA	NA	5	-33.86	78.39	0.8643	0.004048
5	NA	NA	NA	6	-32.81	78.59	1.0568	0.003677
6	NA	-0.3611	NA	6	-32.83	78.62	1.0883	0.003619
7	NA	NA	NA	6	-32.83	78.63	1.0965	0.003605
8	NA	NA	NA	6	-32.97	78.91	1.3837	0.003122
9	NA	NA	-0.3556	6	-33.05	79.06	1.5321	0.002899
10	NA	NA	NA	5	-34.19	79.07	1.5385	0.002890
11	NA	NA	NA	6	-33.07	79.10	1.5751	0.002837
12	-0.33	NA	NA	6	-33.07	79.11	1.5823	0.002827
13	NA	NA	NA	6	-33.14	79.25	1.7232	0.002635
14	NA	NA	NA	6	-33.18	79.32	1.7947	0.002542
15	NA	NA	NA	6	-33.26	79.49	1.9631	0.002337
