# Supplementary material for: Modeling chronic wasting disease transmission risk in mule deer related to habitat characteristics
Source: PLoS One. 2026 Apr 29;21(4):e0346077. doi: 10.1371/journal.pone.0346077 (PMC13127966; doi:10.1371/journal.pone.0346077)
Supplement: S11 Table — Top model included genotype, compound topographic index (CTI) during summer, CTI during winter, and distance to perennial water source during summer. Continuous covariates were standardized prior to model fit. (PDF) [file pone.0346077.s021.pdf]

	**Estimate**	**Std. Error**	**95% Confidence interval**	
(Intercept)	-4.721	1.155	-7.767	-2.884
genotype_categorySS	4.738	1.218	2.755	7.862
scale(ctisum)	1.155	0.414	0.412	2.053
scale(ctiwin)	-1.196	0.422	-2.113	-0.441
scale(mean_dist_pwatersum)	-0.513	0.317	-1.171	0.088
