# Supplementary material for: Modeling chronic wasting disease transmission risk in mule deer related to habitat characteristics
Source: PLoS One. 2026 Apr 29;21(4):e0346077. doi: 10.1371/journal.pone.0346077 (PMC13127966; doi:10.1371/journal.pone.0346077)
Supplement: S12 Table — PRNP genotype was included in all models. A covariate estimate of “NA” indicates the covariate was not included in the specified model. CTI = Compound Topographic Index; KDE = Kernel Density Estimate; RSF = Resource Selection Function. (PDF) [file pone.0346077.s022.pdf]

Model rank	Intercept	Genotype	CTI - summer	CTI - winter	Distance to Cropland - win	Distance to perennial water - summer	Distance to secondary road
1	-5.482	+	1.2072	-0.9981	1.2046	-0.7143	-1.1011
2	-5.122	+	1.5013	-0.8700	1.1679	NA	-0.9938
3	-4.900	+	1.3465	-0.9771	1.0464	NA	-1.0972
4	-5.448	+	1.3166	-0.9110	1.2363	-0.5810	-1.0202
5	-5.482	+	1.2073	-0.9981	1.2046	-0.7144	-1.1015
6	-4.903	+	1.3368	-0.9857	1.0527	NA	-1.0418
7	-5.327	+	0.5941	NA	1.4090	-0.7114	-1.0994
8	-5.376	+	1.0319	NA	1.3939	NA	-0.9505
9	-5.130	+	1.4956	-0.8744	1.1656	NA	-0.9749
10	-5.538	+	0.8447	NA	1.4735	-0.5399	-0.9879
11	-5.453	+	1.3288	-0.9066	1.2426	-0.5916	-1.0505
12	-5.038	+	1.3077	-0.8588	0.7675	NA	NA
13	-5.319	+	1.2277	-0.9047	0.8165	-0.4628	NA
14	-5.213	+	NA	NA	1.3513	-0.8398	-1.0150
15	-5.260	+	1.0697	-1.0279	0.6941	-0.6105	NA

Continued:

Model rank	KDE	RSF quantile	df	logLik	AICc	delta	weight
1	NA	NA	7	-28.16	71.62	0.000	0.18550
2	-0.5736	NA	7	-28.90	73.10	1.476	0.08869
3	NA	NA	6	-30.09	73.15	1.526	0.08648
4	-0.3634	NA	8	-27.79	73.26	1.645	0.08150
5	NA	-0.0007268	8	-28.16	74.01	2.392	0.05610
6	NA	0.1690615	7	-30.01	75.31	3.695	0.02925
7	NA	NA	6	-31.18	75.32	3.703	0.02913
8	-0.7148	NA	6	-31.24	75.44	3.820	0.02747
9	-0.5659	0.0512727	8	-28.89	75.47	3.855	0.02699
10	-0.5498	NA	7	-30.17	75.64	4.021	0.02484
11	-0.3752	-0.0741236	9	-27.77	75.69	4.067	0.02428
12	-0.7203	NA	6	-31.39	75.74	4.117	0.02367
13	-0.5851	NA	7	-30.49	76.29	4.667	0.01799
14	NA	NA	5	-32.81	76.30	4.682	0.01785
15	NA	NA	6	-31.72	76.40	4.777	0.01702
