# Supplementary material for: Modeling chronic wasting disease transmission risk in mule deer related to habitat characteristics
Source: PLoS One. 2026 Apr 29;21(4):e0346077. doi: 10.1371/journal.pone.0346077 (PMC13127966; doi:10.1371/journal.pone.0346077)
Supplement: S13 Table — Top model included genotype, distance to cropland during winter, distance to secondary road, distance to perennial water source during summer, compound topographic index (CTI) during summer, and CTI during winter. Continuous covariates were standardized prior to model fit. (PDF) [file pone.0346077.s023.pdf]

	Estimate	Std. Error	95% Confidence interval	
(Intercept)	-5.4819	1.3307	-8.865	-3.376
genotype_categorySS	5.7432	1.4538	3.391	9.345
scale(mean_dist_cropwin)	1.2046	0.4639	0.345	2.208
scale(mean_dist_road_sec)	-1.1011	0.4710	-2.151	-0.269
scale(mean_dist_pwatersum)	-0.7143	0.3806	-1.516	-0.002
scale(ctisum)	1.2072	0.5048	0.327	2.347
scale(ctiwin)	-0.9981	0.4539	-1.994	-0.187
