# Supplementary material for: Modeling chronic wasting disease transmission risk in mule deer related to habitat characteristics
Source: PLoS One. 2026 Apr 29;21(4):e0346077. doi: 10.1371/journal.pone.0346077 (PMC13127966; doi:10.1371/journal.pone.0346077)
Supplement: S1 File — This file includes additional details on sources of spatial data used as covariates in the resource selection function and risk model analyses. This file also describes the data processing steps in detail. (PDF) [file pone.0346077.s024.pdf]

S1 File. Spatial data details and processing steps.

This file includes additional details on sources of spatial data used as covariates in the resource selection function and risk model analyses. This file also describes the data processing steps in detail.

Any use of trade, firm, or product names is for descriptive purposes only and does not imply endorsement by the U.S. Government.

Erica M. Christensen[1,#a], Nathan J. Kleist[1], David R. Edmunds[1], Julie A. Heinrichs[2], D. Joanne Saher[2], Ashley L. Whipple[1], Melia DeVivo[3,#b], Cameron L. Aldridge[1]

[1]U.S. Geological Survey, Fort Collins Science Center, Fort Collins, Colorado, United States of America

[2]Natural Resource Ecology Laboratory, Colorado State University, Colorado, United States of America

[3]Department of Veterinary Sciences, University of Wyoming, Laramie, Wyoming, United States of America

[#a]Current Address: Department of Fish, Wildlife, and Conservation Ecology, New Mexico State University, Las Cruces, New Mexico, United States of America

[#b]Current Address: Washington Department of Fish and Wildlife, Spokane Valley, Washington, United States of America

Spatial data processing

We chose a set of environmental conditions that we expected were important for mule deer in our area of interest. Below, we provide descriptions of the datasets, grouped into categories of agriculture, human development, water sources, topography, soil, and vegetation. Most datasets were static, meaning they had one timestamp, except for the Rangeland Condition Monitoring Assessment and Projection (RCMAP), Rangeland Analysis Platform (RAP), USDA-NASS Crop Data Layer (CDL), and IrrMapper datasets, which had yearly timestamped layers that we matched to our GPS point locations (2010-2014). All datasets were reprojected to Albers Conical Equal Area (WGS 1984) and cropped to our study area extent prior to analysis. To create the study area shape, we created a 100% minimum convex polygon (MCP) around the full dataset of GPS points and then buffered the MCP by 2,548 m. All spatial manipulations described below were executed in Pycharm (2021.3.2, Community edition) using the ArcPy site package and the ArcGIS Spatial Analyst extension tool (Copyright 2024 Esri Inc., ArcGIS Pro: 3.4.3.).

Agricultural data

We used the USDA-NASS Crop Data Layer (CDL) to estimate Euclidean distance in meters (m) and proportion of cropland within buffers for each year 2010-2014 (USDA-NASS 2020). We

categorized the CDL into “cropland” and “non-cropland.” The following non-cropland categories were recategorized to equal a value of “0” for proportion and “NoData” for distance estimates, all other categories were considered “cropland” and were recategorized to equal a value of “1;” Other Hay/Non Alfalfa, Fallow/Idle Cropland, Forest, Shrubland, Barren, Clouds/No Data, Developed, Water, Wetlands, Nonag/Undefined, Aquaculture, Open Water, Perennial Ice/Snow, Developed/Open Space, Developed/Low Intensity, Developed/Med Intensity, Developed/High Intensity, Deciduous Forest, Evergreen Forest, Mixed Forest, Shrubland, Grassland/Pasture, Woody Wetlands, Herbaceous Wetlands. To identify irrigated lands we used the IrrMapper dataset (Ketchum et al. 2020) for each year 2010-2014. Using the IrrMapper raster (1 = irrigated, NoData = non-irrigated) we calculated the Euclidean distance (m) of each 30-m pixel to the nearest irrigated pixel.

Human development data

To identify roads, we used the TIGER dataset (U.S. Census Bureau 2019) and distinguished line segments representing primary roads (MTFCC code of S1100), secondary roads (MTFCC code of S1200), and local and 4-wheel-drive (4WD) roads (MTFCC code of S1400 or S1500). We created distance rasters to capture the Euclidean distance (m) of each 30-m pixel to each road feature (primary, secondary, or local/4WD) as well as a density raster to capture road density of secondary roads only (kilometers [km] of road within 6.4km) (km/km^2). We estimated time spent in developed land covers by extracting values from deer GPS points that overlapped NLCD 2021 (Dewitz 2023) land cover classes of low, medium, and/or high intensity development and then calculated the percent of each deer’s yearly use points spent in the combined “developed” category.

Water source data

We represented distance to water features using the USGS National Hydrography Dataset (NHD; USGS 2022). We subset the dataset to represent five categories of water features, 1) perennial lake/pond or reservoir (FCODE = 39004, 39009, 39010, 39011, 39012, 43615, or 43621) plus lake/ponds and reservoirs that were designated a null hydrographic category (FCODE = 39000 or 43600, respectively), 2) intermittent lake/pond or reservoir (FCODE = 39001, 39005, 39006, or 43614), 3) perennial stream/river (FCODE = 46006, plus the North Platte River), 4) ephemeral stream/river (FCODE = 46007), and 5) springs and seeps (FType = 458). We also created a feature layer that combined all perennial water sources (spring/seep, lake/pond, reservoir, stream/river). We created distance rasters to capture the Euclidean distance (m) of each 30-m pixel to each water feature category. We estimated time spent in wetlands by extracting values from deer GPS points that overlapped NLCD 2021 land cover classes of woody wetlands and/or emergent herbaceous wetlands and then calculated the percent of each deer’s yearly use points spent in the combined “wetland” category.

Topography data

We used the digital elevation model (DEM), vector ruggedness measure (VRM), compound topographic index (CTI), and heat load index (HLI) layers described in the supplement of O'Donnell et al. 2022. VRM represents a decomposition of slope and aspect into 3-dimensonal vectors summarized within a 1000-m focal area, which provides a measure of terrain ruggedness, independent of slope. CTI represents a steady-state wetness, a function of slope and upstream contributing area per unit width that is orthogonal to the flow direction (i.e., ratio of catchment area and slope). Flatter areas will have large values (typically not informative), while smaller catchments with steep slopes will reflect low values of the index. HLI identifies the potential annual direct incident radiation suitable for our latitudes of 30 – 60 degrees. We derived aspect from the DEM (O'Donnell et al. 2022) using the Surface Parameters tool in ArcGIS Pro 3.3.1 which fit a quadratic surface function over a 3x3 neighborhood. We then converted aspect (in degrees) to a continuous scale from 0 to 1 to represent the extent of southern exposure (0=north; 1=south) using the equation: [1-cos([2(pi)*aspect]/360)]/2 (Cutler et al. 2007).

Soil data
Soil properties (percent sand, percent clay, pH, and organic carbon) at 100 m resolution were obtained from Ramcharan et al. (2018). Data were available at multiple soil depths, and we chose surface depth (0 cm) since mule deer are most likely to interact with the surface layer. Annual average soil moisture was obtained from O'Donnell and Manier (2022). This data set represents estimates of annual soil moisture at 30 m resolution based on long term climate normals from 1981-2010.

Vegetation data
We used the Rangeland Condition Monitoring Assessment and Projection (RCMAP; Rigge et al. 2024) datasets to represent the mean fractional covers of trees, shrubs, and herbaceous vegetation for each year 2010-2014. We used the Rangeland Analysis Platform (RAP; Allred et al. 2021) to represent mean annual aboveground biomass (pounds/acre) of annual forb/grass and perennial forb/grass for each year 2010-2014. We used the National Land Cover Database (NLCD; Coulston et al. 2012) 2016 tree cover dataset (30-m pixel resolution) to estimate Euclidean distance (m) to trees. We reclassified cover values as follows, 1 = percent cover between 1-100%, and NoData = 0% cover. Euclidean distance was then measured from each 30-m pixel to the closest tree cover (pixel value = 1). To estimate proportion of early growth conifer and pinyon-juniper cover (1-10% cover) we first extracted existing vegetation types (EVT) from Landfire 2016 2.0.0 that represent conifer and/or pinyon-juniper dominated ecological systems. Specifically, we classified the following EVT codes as "1" and everything else as "NoData": 9001, 9062, 7016, 7017, 7019, 7020, 7023, 7025, 7026, 7027, 7049, 7053, 7054, 7057, 7059, 7060, 7101, 7102, 7104, 7114, 7115, 7116, 7118, 7119, 7167, 7179, 7264, 7265. We then extracted tree canopy cover values from the NLCD 2016 tree cover dataset (Coulston et al. 2012) that overlapped pixel values of "1" (conifer/pinyon-juniper vegetation types). Lastly, we reclassified cover values of 1-10% cover to "1" and everything else "0". Proportion of early

growth conifer and pinyon-juniper cover was then estimated within buffers of interest using this binary raster.

References

Allred, B.W., B.T. Bestelmeyer, C.S. Boyd, C. Brown, K.W. Davies, M.C. Duniway, L.M. Ellsworth, T.A. Erickson, S.D. Fuhlendorf, T.V. Griffiths, V. Jansen, M.O. Jones, J. Karl, A. Knight, J.D. Maestas, J.J. Maynard, S.E. McCord, D.E. Naugle, H.D. Starns, D. Twidwell, and D.R. Uden. 2021. Improving Landsat predictions of rangeland fractional cover with multitask learning and uncertainty. Methods in Ecology and Evolution. http://dx.doi.org/10.1111/2041-210x.13564

Coulston, J. W., Moisen, G. G., Wilson, B. T., Finco, M. V., Cohen, W. B., Brewer, C. K., 2012, Modeling percent tree canopy cover—A pilot study: Photogrammetric Engineering and Remote Sensing, v. 78, no. 7, p. 715–727, at https://doi.org/10.14358/PERS.78.7.715

Cutler DR, Edwards Jr. TC, Beard KH, Cutler A, Hess KT, Gibson J, et al. Random Forests for Classification in Ecology. Ecology. 2007;88: 2783–2792. doi:10.1890/07-0539.1

Dewitz, J., 2023, National Land Cover Database (NLCD) 2021 Products: U.S. Geological Survey data release, https://doi.org/10.5066/P9JZ7AO3

Ketchum, D.; Jencso, K.; Maneta, M.P.; Melton, F.; Jones, M.O.; Huntington, J. IrrMapper: A Machine Learning Approach for High Resolution Mapping of Irrigated Agriculture Across the Western U.S.. Remote Sens. 2020, 12, 2328. doi:10.3390/rs12142328.

O'Donnell, M. S., D. R. Edmunds, C. L. Aldridge, J. A. Heinrichs, A. P. Monroe, P. S. Coates, B. G. Prochazka, S. E. Hanser, and L. A. Wiechman. 2022. Defining biologically relevant and hierarchically nested population units to inform wildlife management. Ecology and Evolution 12:22, doi: 10.1002/ece3.9565.

O'Donnell MS, Manier DJ (2022) Soil-climate estimates in the western United States: climate averages (1981–2010): U.S. Geological Survey data release. https://doi.org/10.5066/P9ULGC03

Ramcharan A., Hengl T., Nauman T., Brungard C., Waltman S., Wills S., Thompson J. 2018. Soil Property and Class Maps of the Conterminous US at 100 meter Spatial Resolution based on a Compilation of National Soil Point Observations and Machine Learning. Soil Science Society of America Journal. https://doi.org/10.2136/sssaj2017.04.0122

Rigge, M.B., Bunde, B., Postma, K., and Shi, H., 2024, Rangeland Condition Monitoring Assessment and Projection (RCMAP) Fractional Component Time-Series Across the Western U.S. 1985-2023: U.S. Geological Survey data release, https://doi.org/10.5066/P9SJXUI1

U.S. Census Bureau, 2019 TIGER/Line Shapefiles, 2019, https://www.census.gov/geographies/mapping-files/time-series/geo/tiger-geodatabase-file.2019.html, accessed on June 02, 2020.

USDA-NASS. 2020. Cropland Data Layer (CDL), 2020 edition. Published crop-specific raster data layer. USDA-NASS, Washington, D.C. Available: https://nassgeodata.gmu.edu/CropScape/ [Accessed 12 May 2022].

U.S. Geological Survey, National Geospatial Program, 2022, USGS National Hydrography in FileGDB 10.1 format (published 20220227): U.S. Geological Survey. https://www.sciencebase.gov/catalog/item/5ea068ae82cefae35a12a120
