# Supplementary material for: Modeling chronic wasting disease transmission risk in mule deer related to habitat characteristics
Source: PLoS One. 2026 Apr 29;21(4):e0346077. doi: 10.1371/journal.pone.0346077 (PMC13127966; doi:10.1371/journal.pone.0346077)
Supplement: S2 File — This file includes additional details on methods and results of the resource selection function analysis done to support the manuscript “Modeling chronic wasting disease transmission risk in mule deer related to habitat characteristics.”. (PDF) [file pone.0346077.s025.pdf]

S2: Resource selection function methods

This file includes additional details on methods of the resource selection function analysis done to support the manuscript "Modeling chronic wasting disease transmission risk in mule deer related to habitat characteristics."

Any use of trade, firm, or product names is for descriptive purposes only and does not imply endorsement by the U.S. Government.

Erica M. Christensen[1,#a], Nathan J. Kleist[1], David R. Edmunds[1], Julie A. Heinrichs[2], D. Joanne Saher[2], Ashley L. Whipple[1], Melia DeVivo[3,#b], Cameron L. Aldridge[1]

[1]U.S. Geological Survey, Fort Collins Science Center, Fort Collins, Colorado, United States of America

[2]Natural Resource Ecology Laboratory, Colorado State University, Colorado, United States of America

[3]Department of Veterinary Sciences, University of Wyoming, Laramie, Wyoming, United States of America

[#a]Current Address: Department of Fish, Wildlife, and Conservation Ecology, New Mexico State University, Las Cruces, New Mexico, United States of America

[#b]Current Address: Washington Department of Fish and Wildlife, Spokane Valley, Washington, United States of America

Methods

As an alternative measure of deer habitat use, we used an RSF framework to evaluate mule deer resource selection as a function of landscape variables using binomial generalized linear models (GLMs) in R (version 4.4.2; R Core Team 2024). Because our goal for these RSF models was to produce the most predictive models possible, we did not strive for parsimony. Separate models were constructed for non-migratory deer, migratory deer on summer range, and migratory deer on winter range. Migratory status for each deer was determined by visually inspecting the GPS record for each individual. We classed successive movements in the same direction that resulted in a spatially separate seasonal range as migratory. GPS point data was used from a larger number of individuals than was used in the risk models and included both male and female deer. Deer that did not have at least 3 months of tracking data were dropped because they could not be reliably categorized as migratory or nonmigratory. GPS use points with a dilution of precision of less than 4 were dropped and the remaining points were down sampled to one randomly selected point per individual per day in order to reduce the effect of spatial autocorrelation, resulting in 53,787 use points. We included use points from deer regardless of CWD status; we could not find evidence in the literature to suggest that CWD positive and negative deer select different habitats except for CWD+ deer in the final clinical stages of the disease (i.e., the last few weeks

of life). Since we down-sampled to one GPS point per deer per day, any potential points from CWD clinical deer represented a small percentage of the total number of use points, and likely had a negligible effect on results. The final migratory status groups for modeling were migratory summer, migratory winter, and non-migratory. The migratory winter dataset comprised a total of 58 unique individuals (56 females, 2 males), which were split into a training set of 44 individuals (43 females, 1 male) and a testing set of 14 individuals (13 females, 1 male). The migratory summer dataset consisted of 54 unique individuals (52 females, 2 males), divided into a training set of 42 individuals (41 females, 1 male) and a testing set of 12 individuals (11 females, 1 male). Lastly, the non-migratory dataset included 46 unique individuals (33 females, 13 males), partitioned into a training set of 36 individuals (25 females, 11 males) and a testing set of 10 individuals (8 females, 2 males).

The sampling area for the purpose of selecting available points was defined by constructing a 100% minimum convex polygon (MCP) around the full dataset of GPS points and applying a buffer of 48,310 m. The buffer represents the maximum distance traveled by any one deer in our study. We evaluated beta stability across different used to available point ratios ranging from 1:1 to 1:24 (used:available). The ratio 1:15 produced beta stability the most consistently and was used in the RSF modeling framework (n = 806,805 available points).

Environmental variables used as covariates in the RSF included measures related to agriculture, human development, water sources, topography, and vegetation cover. Specifically, we tested for effects of distance to cropland (USDA-NASS 2020), distance to irrigated land (Ketchum et al. 2020), proportion of area within a buffer of used/available points designated as cropland (USDA-NASS 2020), distance to nearest primary road (i.e., interstate highways) (U.S. Census Bureau 2019), distance to nearest secondary road (i.e., state highways) (U.S. Census Bureau 2019), density of secondary roads (U.S. Census Bureau 2019), distance to nearest local or 4-wheel-drive road (U.S. Census Bureau 2019), distance to nearest spring/seep (USGS NHD 2022), distance to perennial lake/pond/reservoir (USGS NHD 2022), distance to perennial stream/river (USGS NHD 2022), distance to ephemeral stream/river (USGS NHD 2022), distance to intermittent lake/pond/reservoir (USGS NHD 2022), heat load index (HLI) derived from elevation using methods from McCune and Keon 2002, vector ruggedness measure (VRM) derived from elevation using methods from (Sappington et al. 2007), compound topographic index (CTI) derived from elevation using methods from (Gessler et al. 1995), aspect derived from elevation and standardized using methods from (Cutler et al. 2007), distance to nearest trees (where tree cover >1%) (NLCD TCC; Coulston et al. 2012), percent tree cover (RCMAP; Rigge et al. 2024)), percent shrub cover (RCMAP; Rigge et al. 2024), annual vegetation biomass (RAP; Jones et al. 2021), perennial vegetation biomass (RAP; Jones et al. 2021), and proportion of area within a buffer of used/available points designated as early growth (1-10% canopy cover) of conifer/pinyon-juniper (LANDFIRE 2016).

We investigated mule deer habitat use over a range of spatial scales and selected the most informative to use in our candidate models. We examined scales of 0 m (direct intersect of 30x30m pixel value), 50 m, 100 m, 250 m, 500 m, 100 m, 1515 m (average daily movement distance), and 2548 m (diameter of average range size) circular moving windows. We evaluated the mean environmental variable condition over each moving window radius. To investigate the effect of proximity to certain features (i.e. roads, rivers, agriculture), we calculated both Euclidean and distance decay functions, using the scale sizes to determine the shape of the

decay. We also considered linear density of secondary roads over each scale as a possible predictor of mule deer habitat use. We evaluated whether quadratic forms of four variables: density of secondary roads, distance to cropland, percent tree cover, and percent shrub cover were better predictors than their linear forms. Because of the amount of location data available to us for modeling, quadratic forms of variables are almost always selected over linear forms, therefore we restricted our investigation into non-linear relationships to those variables which we considered to be ecologically defensible.

We competed predictor variables with the same base against each other (form and scale) by fitting univariable models, and selected the optimal scale for each variable using Akaike's Information Criterion for small sample sizes (AICc) (Burnham and Anderson 2002). After selecting the optimal predictor for each habitat covariate, we tested for high correlation between covariates. We removed variables with correlations > 0.70, or selected another highly-ranked scale for a correlated covariate. Selected variables were retained (form and scale) throughout the modeling process unless modeling requirements necessitated changes (see below).

We split data into training (80%) and testing (20%) sets for each seasonal model. We assessed seasonal models using least absolute shrinkage and selection operator (LASSO) using the cv.glmnet() function in the glmnet R package (Friedman et al. 2010). We selected the regularization penalty at the 1 standard error threshold. Results suggested removing some first-order polynomials, but these were retained because they were components of quadratic terms—generated via the function 'step_poly()' from the 'tidymodels' collection of R packages (Kuhn and Wickham 2020)—for which the second-order polynomials were not suggested for removal. There was otherwise no evidence to remove any variables as the full model had the lowest deviance. For the final models, we scaled $(x - \bar{x}/sd)$ all habitat predictors and applied infinite weights to available points. We addressed any warnings of "near perfect separation" in model fitting by selecting a different scale for some covariates. For example, distance to primary road with short-distance decay had a coefficient near 100 while other coefficients were near 1, and so we included distance to primary road with a longer distance decay.

We then used the final models to predict relative probability of habitat use and applied the models spatially across the sampling area (MCP) and a larger area of interest (AOI) using the 'predict' function in the 'terra' package (Hijmans 2024). The larger AOI was created by extending the MCP out by the maximum distance travelled by any one mule deer in the study, which was 48,310 m. Predictions were generated on the logit scale, and we subtracted the model-specific intercept to obtain intercept-corrected logit values, which were then exponentiated to derive the raw "Probability of Use." To standardize these predicted probabilities, we divided each by the maximum probability so that the resulting relative probabilities spanned from 0 to 1. We then classified these relative probabilities within the MCP into 20 quantile bins. This process of creating relative probability quantiles was conducted separately for each migratory group dataset. Finally, using the ranges of the MCP quantile bins, we applied these quantile thresholds to the larger AOI surface to maintain consistency. We assessed model fit by extracting expected quantile bins from the predicted surfaces at the locations of an independent testing dataset. For the nonmigratory model, 99% of the testing points fell within the top four quantile bins; for the migratory summer model, 60% of the testing points were located in the top four quantile bins; and for the migratory winter model, 92% of the testing points were found in the top four quantile bins.

References

Burnham KP, Anderson DR. Model Selection and Multimodel Inference: A Practical Information-Theoretic Approach. 2nd edition. Springer-Verlag, New York; 2002.

Coulston, J. W., Moisen, G. G., Wilson, B. T., Finco, M. V., Cohen, W. B., Brewer, C. K., 2012, Modeling percent tree canopy cover—A pilot study: Photogrammetric Engineering and Remote Sensing, v. 78, no. 7, p. 715–727, at https://doi.org/10.14358/PERS.78.7.715

Cutler DR, Edwards Jr. TC, Beard KH, Cutler A, Hess KT, Gibson J, et al. Random Forests for Classification in Ecology. Ecology. 2007;88: 2783–2792. doi:10.1890/07-0539.1

Friedman, J., T. Hastie, and R. Tibshirani (2010). Regularization Paths for Generalized Linear Models via Coordinate Descent. Journal of Statistical Software_, 33(1), 1-22. doi:10.18637/jss.v033.i01.

Gessler, P.E., Moore, I.D., McKenzie, N.J., and Ryan, P.J. (1995) Soil-landscape modelling and spatial prediction of soil attributes, International Journal of Geographical Information Systems, 9:4, 421-432, https://doi.org/10.1080/02693799508902047

Hijmans R (2024). _terra: Spatial Data Analysis_. R package version 1.7-71, <https://CRAN.R-project.org/package=terra>.

Jones MO, Robinson NP, Naugle DE, Maestas JD, Reeves MC, Lankston RW, et al. Annual and 16-Day Rangeland Production Estimates for the Western United States. Rangeland Ecology & Management. 2021;77: 112–117. doi:10.1016/j.rama.2021.04.003

Ketchum, D.; Jencso, K.; Maneta, M.P.; Melton, F.; Jones, M.O.; Huntington, J. IrrMapper: A Machine Learning Approach for High Resolution Mapping of Irrigated Agriculture Across the Western U.S.. Remote Sens. 2020, 12, 2328. doi:10.3390/rs12142328.

Kuhn, M., and H. Wickham. 2020. "Tidymodels: A Collection of Packages for Modeling and Machine Learning Using tidyverse Principles."

LANDFIRE, Earth Resources Observation and Science Center (EROS), U.S. Geological Survey. 2016. LANDFIRE Remap 2016 Existing Vegetation Type (EVT) CONUS. Accessed 8/11/2020. https://www.landfire.gov.

McCune, B., & Keon, D. (2002). Equations for potential annual direct incident radiation and heat load. Journal of Vegetation Science, 13(4), 603-606. doi:10.1111/j.1654-1103.2002.tb02087.x

R Core Team. 2024. R: A language and environment for statistical computing. R Foundation for Statistical Computing, Vienna, Austria.

Rigge, M.B., Bunde, B., Postma, K., and Shi, H., 2024, Rangeland Condition Monitoring Assessment and Projection (RCMAP) Fractional Component Time-Series Across the Western U.S. 1985-2023: U.S. Geological Survey data release, https://doi.org/10.5066/P9SJXUI1

Sappington, J.M., K.M. Longshore, D.B. Thomson (2007). Quantifying Landscape Ruggedness for Animal Habitat Analysis: A case Study Using Bighorn Sheep in the Mojave Desert. Journal of Wildlife Management. 71(5):1419-1426, https://doi.org/10.2193/2005-723

U.S. Census Bureau, 2019 TIGER/Line Shapefiles, 2019, https://www.census.gov/geographies/mapping-files/time-series/geo/tiger-geodatabase-file.2019.html, accessed on June 02, 2020.

USDA-NASS. 2020. Cropland Data Layer (CDL), 2020 edition. Published crop-specific raster data layer. USDA-NASS, Washington, D.C. Available: https://nassgeodata.gmu.edu/CropScape/ [Accessed 12 May 2022].

U.S. Geological Survey, National Geospatial Program, 2022, USGS National Hydrography in FileGDB 10.1 format (published 20220227): U.S. Geological Survey. https://www.sciencebase.gov/catalog/item/5ea068ae82cefae35a12a120
