# Supplementary material for: Modeling chronic wasting disease transmission risk in mule deer related to habitat characteristics
Source: PLoS One. 2026 Apr 29;21(4):e0346077. doi: 10.1371/journal.pone.0346077 (PMC13127966; doi:10.1371/journal.pone.0346077)
Supplement: Attachment — Submitted filename: PONE_Response_to_reviewers.docx [file pone.0346077.s027.docx]

PONE-D-25-40590

Modeling chronic wasting disease transmission risk in mule deer related to habitat characteristics

PLOS ONE

Journal Requirements:

When submitting your revision, we need you to address these additional requirements.

1. Please ensure that your manuscript meets PLOS ONE's style requirements, including those for file naming. The PLOS ONE style templates can be found at https://journals.plos.org/plosone/s/file?id=wjVg/PLOSOne_formatting_sample_main_body.pdf and <https://journals.plos.org/plosone/s/file?id=ba62/PLOSOne_formatting_sample_title_authors_affiliations.pdf>

We have adjusted the manuscript and S1 Supplement to meet style requirements.

2. Please include your full ethics statement in the ‘Methods’ section of your manuscript file. In your statement, please include the full name of the IRB or ethics committee who approved or waived your study, as well as whether or not you obtained informed written or verbal consent. If consent was waived for your study, please include this information in your statement as well.

No original data were collected for this study, so there is no study-specific IRB or ethics committee information to report. Data collection methods and approvals were reported in the original study publication (DeVivo et al. 2017), which we have repeated in the Methods section of our manuscript for informational purposes.

- DeVivo MT, Edmunds DR, Kauffman MJ, Schumaker BA, Binfet J, Kreeger TJ, et al. Endemic chronic wasting disease causes mule deer population decline in Wyoming. PLOS ONE. 2017;12: e0186512. doi:10.1371/journal.pone.0186512

3. In this instance it seems there may be acceptable restrictions in place that prevent the public sharing of your minimal data. However, in line with our goal of ensuring long-term data availability to all interested researchers, PLOS’ Data Policy states that authors cannot be the sole named individuals responsible for ensuring data access (http://journals.plos.org/plosone/s/data-availability#loc-acceptable-data-sharing-methods).

   Data requests to a non-author institutional point of contact, such as a data access or ethics committee, helps guarantee long term stability and availability of data. Providing interested researchers with a durable point of contact ensures data will be accessible even if an author changes email addresses, institutions, or becomes unavailable to answer requests.

   Before we proceed with your manuscript, please also provide non-author contact information (phone/email/hyperlink) for a data access committee, ethics committee, or other institutional body to which data requests may be sent. If no institutional body is available to respond to requests for your minimal data, please consider if there any institutional representatives who did not collaborate in the study, and are not listed as authors on the manuscript, who would be able to hold the data and respond to external requests for data access? If so, please provide their contact information (i.e., email address). Please also provide details on how you will ensure persistent or long-term data storage and availability.

We have added a citation to a portion of the data which is stored on Dryad: “Data on each deer’s sex, genotype, age at first capture, CWD test result at first capture, date of last test with CWD not detected, and date of first positive CWD test result (if applicable) are publicly available on Dryad (DeVivo et al. 2018).” (line 170 in tracked changes document).

The GPS locations are sensitive information and cannot be made publicly available, but the data owner has uploaded the data to the Movebank repository. We have added the following sentence to the methods citing this: “The GPS locations are available by request from Movebank (movebank.org, study name “Chronic Wasting Disease Ecology and Epidemiology of Mule Deer in Wyoming,” study ID 7727287671).” (line 173 in tracked changes document).

DeVivo MT, Edmunds DR, Kauffman MJ, Schumaker BA, Binfet J, Kreeger TJ, et al. Data from: Endemic chronic wasting disease causes mule deer population decline in Wyoming. Dryad; 2018. p. 51623 bytes. doi:10.5061/DRYAD.H66CN

4. When completing the data availability statement of the submission form, you indicated that you will make your data available on acceptance. We strongly recommend all authors decide on a data sharing plan before acceptance, as the process can be lengthy and hold up publication timelines. Please note that, though access restrictions are acceptable now, your entire data will need to be made freely accessible if your manuscript is accepted for publication. This policy applies to all data except where public deposition would breach compliance with the protocol approved by your research ethics board. If you are unable to adhere to our open data policy, please kindly revise your statement to explain your reasoning and we will seek the editor's input on an exemption. Please be assured that, once you have provided your new statement, the assessment of your exemption will not hold up the peer review process.

We have created a USGS data release where the final raster maps resulting from these analyses are publicly available. We have added the citation to this data release (line 653 in tracked changes document, the data can be found here: doi.org/10.5066/P1K3QFC8).

5. We note that Figure 1 in your submission contain map images which may be copyrighted. All PLOS content is published under the Creative Commons Attribution License (CC BY 4.0), which means that the manuscript, images, and Supporting Information files will be freely available online, and any third party is permitted to access, download, copy, distribute, and use these materials in any way, even commercially, with proper attribution. For these reasons, we cannot publish previously copyrighted maps or satellite images created using proprietary data, such as Google software (Google Maps, Street View, and Earth). For more information, see our copyright guidelines: <http://journals.plos.org/plosone/s/licenses-and-copyright>. We require you to either (a) present written permission from the copyright holder to publish these figures specifically under the CC BY 4.0 license, or (b) remove the figures from your submission:
   a. You may seek permission from the original copyright holder of Figure 1 to publish the content specifically under the CC BY 4.0 license.  We recommend that you contact the original copyright holder with the Content Permission Form (http://journals.plos.org/plosone/s/file?id=7c09/content-permission-form.pdf) and the following text: “I request permission for the open-access journal PLOS ONE to publish XXX under the Creative Commons Attribution License (CCAL) CC BY 4.0 (http://creativecommons.org/licenses/by/4.0/). Please be aware that this license allows unrestricted use and distribution, even commercially, by third parties. Please reply and provide explicit written permission to publish XXX under a CC BY license and complete the attached form.” Please upload the completed Content Permission Form or other proof of granted permissions as an ""Other"" file with your submission. In the figure caption of the copyrighted figure, please include the following text: “Reprinted from [ref] under a CC BY license, with permission from [name of publisher], original copyright [original copyright year].”
   b. If you are unable to obtain permission from the original copyright holder to publish these figures under the CC BY 4.0 license or if the copyright holder’s requirements are incompatible with the CC BY 4.0 license, please either i) remove the figure or ii) supply a replacement figure that complies with the CC BY 4.0 license. Please check copyright information on all replacement figures and update the figure caption with source information. If applicable, please specify in the figure caption text when a figure is similar but not identical to the original image and is therefore for illustrative purposes only. The following resources for replacing copyrighted map figures may be helpful: USGS National Map Viewer (public domain): <http://viewer.nationalmap.gov/viewer/>; The Gateway to Astronaut Photography of Earth (public domain): <http://eol.jsc.nasa.gov/sseop/clickmap/>; Maps at the CIA (public domain): https://www.cia.gov/library/publications/the-world-factbook/index.html and <https://www.cia.gov/library/publications/cia-maps-publications/index.html> NASA Earth Observatory (public domain): <http://earthobservatory.nasa.gov/> Landsat: <http://landsat.visibleearth.nasa.gov/> USGS EROS (Earth Resources Observatory and Science (EROS) Center) (public domain): [http://eros.usgs.gov/#](http://eros.usgs.gov/) Natural Earth (public domain): <http://www.naturalearthdata.com/>

Figure 1 does not contain any copyrighted images. It was generated entirely by the authors using R, using publicly available data for elevation, U.S. state outlines, and locations of cities, rivers, and roads. We have added citations to data sources and software packages to the Figure 1 caption: “Data for elevation, road, and river were obtained from publicly available data sources (U.S. Census Bureau 2019, U.S. Geological Survey 2022, U.S. Geological Survey 2018). Map was created using R software (R Core Team 2025) including packages ‘ggplot2,’ ‘ggspatial,’ ‘maps,’ ‘cowplot,’ and ‘terra’ (Becker et al. 2025, Wickham 2016, Hijmans 2025, Dunnington 2025, and Wilke 2025).”

U.S. Census Bureau. TIGER/Line Shapefiles. 2019. Available:	<https://www.census.gov/geographies/mapping-files/time-series/geo/tiger->	geodatabase-file.2019.html

U.S. Geological Survey. USGS National Hydrography in FileGDB 10.1 format. 2022.	Available: https://www.sciencebase.gov/catalog/item/5ea068ae82cefae35a12a120

U.S. Geological Survey. 1/3rd arc-second digital elevation models (DEMs) - USGS National	Map 3DEP downloadable data collection. 2018. Available:	<https://www.usgs.gov/core-science-systems/ngp/3dep/about-3dep-products->	services

R Core Team. R: A language and environment for statistical computing. Vienna, Austria: R	Foundation for Statistical Computing; 2024. Available: <https://www.R-project.org/>

Becker RA, Wilks AR, Brownrigg R, Minka TP, Deckmyn A. maps: Draw Geographical Maps.	2025. doi:10.32614/CRAN.package.maps

Wickham H. ggplot2: Elegant Graphics for Data Analysis. Springer-Verlag New York; 2016.	Available: https://ggplot2.tidyverse.org

Hijmans RJ. terra: Spatial Data Analysis. 2025. doi:10.32614/CRAN.package.terra

Dunnington D. ggspatial: Spatial Data Framework for ggplot2. 2025.	doi:10.32614/CRAN.package.ggspatial

Wilke CO. cowplot: Streamlined Plot Theme and Plot Annotations for ggplot2. 2025.	doi:10.32614/CRAN.package.cowplot

6. Please include captions for your Supporting Information files at the end of your manuscript, and update any in-text citations to match accordingly. Please see our Supporting Information guidelines for more information: <http://journals.plos.org/plosone/s/supporting-information>.

We have renamed the Supplement to “S1 Supplement” and renamed supplemental tables and figures (e.g., “Table A1 in S1 Supplement”) in order to conform to the guidelines. We have also added the captions for figures and tables from the S1 Supplement at the end of the manuscript.

7. Please remove all personal information, ensure that the data shared are in accordance with participant consent, and re-upload a fully anonymized data set. Note: spreadsheet columns with personal information must be removed and not hidden as all hidden columns will appear in the published file. Additional guidance on preparing raw data for publication can be found in our Data Policy (https://journals.plos.org/plosone/s/data-availability#loc-human-research-participant-data-and-other-sensitive-data) and in the following article: <http://www.bmj.com/content/340/bmj.c181.long>.

We have ensured that no personal information appears in the submitted files.

8. If the reviewer comments include a recommendation to cite specific previously published works, please review and evaluate these publications to determine whether they are relevant and should be cited. There is no requirement to cite these works unless the editor has indicated otherwise.

The reviewers have not indicated any specific citation recommendations.

9. Please review your reference list to ensure that it is complete and correct. If you have cited papers that have been retracted, please include the rationale for doing so in the manuscript text, or remove these references and replace them with relevant current references. Any changes to the reference list should be mentioned in the rebuttal letter that accompanies your revised manuscript. If you need to cite a retracted article, indicate the article’s retracted status in the References list and also include a citation and full reference for the retraction notice.
   We have reviewed the reference list for completeness and to conform to formatting guidelines. We have not identified any retracted articles.

--------------------------------------------------------------------------------------------------------------

Reviewers' comments:

Reviewer's Responses to Questions

**Comments to the Author**

1. Is the manuscript technically sound, and do the data support the conclusions?

The manuscript must describe a technically sound piece of scientific research with data that supports the conclusions. Experiments must have been conducted rigorously, with appropriate controls, replication, and sample sizes. The conclusions must be drawn appropriately based on the data presented.

Reviewer #1: Yes

Reviewer #2: Yes

2. Has the statistical analysis been performed appropriately and rigorously?

Reviewer #1: Yes

Reviewer #2: Yes

3. Have the authors made all data underlying the findings in their manuscript fully available?

The [PLOS Data policy](http://www.plosone.org/static/policies.action#sharing) requires authors to make all data underlying the findings described in their manuscript fully available without restriction, with rare exception (please refer to the Data Availability Statement in the manuscript PDF file). The data should be provided as part of the manuscript or its supporting information, or deposited to a public repository. For example, in addition to summary statistics, the data points behind means, medians and variance measures should be available. If there are restrictions on publicly sharing data—e.g. participant privacy or use of data from a third party—those must be specified.

Reviewer #1: Yes

Reviewer #2: No

4. Is the manuscript presented in an intelligible fashion and written in standard English?

PLOS ONE does not copyedit accepted manuscripts, so the language in submitted articles must be clear, correct, and unambiguous. Any typographical or grammatical errors should be corrected at revision, so please note any specific errors here.

Reviewer #1: Yes

Reviewer #2: Yes

5. Review Comments to the Author

Please use the space provided to explain your answers to the questions above. You may also include additional comments for the author, including concerns about dual publication, research ethics, or publication ethics. (Please upload your review as an attachment if it exceeds 20,000 characters)

------------------------------------------------------------------------------------------------------------------

Reviewer #1: Manuscript Number PONE-D-25-40590
Title: Modeling chronic wasting disease transmission risk related to habitat
Characteristics.

The authors have conducted a challenging study developing predictive models of CWD indirect transmission in mule deer using data from GPS-collared animals tested for CWD in Wyoming. Challenges associated with CWD and its impact on cervids continue to require an integration of field, laboratory, and biostatistical approaches to address disease expansion, persistence, surveillance, and control strategies.
This study modeled the probability of mule deer acquiring CWD (new infections) while accounting for variables that may impact indirect transmission of CWD. It accounted for the environmental properties of the study area that facilitate prion persistence, as well as for animal genotype, age, migratory status, and covariates associated with deer density and habitat characteristics used by the fifty-nine mule deer in the study. The best models did not include deer density, but they did include habitat suitability and environmental properties associated with prion retention. The study revealed differences in the risk of CWD in the landscape. Furthermore, PRNP genotype, but not age or migratory status, influenced the probability of contracting CWD, and habitat selection alone was unable to explain CWD risk in its entirety.

The manuscript was well written, provided detailed descriptions of the process, logical transitions, and a clear presentation of the mathematical models and results. I enjoyed reading the manuscript and going over the supplementary material.

We thank the reviewer for their comments, and we have responded to their suggestions below:

My only recommendations are:
1) Add "mule deer" to the title.

This has been added.
2) Reference figures 2, 3, and 4 in the text.

Fig. 2-3 are referenced on line 619 and 732, Fig. 4 is referenced on line 647 and 650 in the tracked changes document.
3) To add to the figures (2,3 &4) or to the discussion/conclusion, an example of how wildlife managers can use this work. That is, help them understand how to apply this work. After all, the abstract concludes: with one of the applications of this work as these “spatially varying risk factors can support managers in designing data collection and disease management strategies.”

Thank you for this suggestion. We have added the following to the discussion (line 829 of tracked changes document): “For example, combining our approach to identify potential environmental reservoirs of CWD with new methods currently being developed to detect CWD prions in soils (Katircioglu 2025) or use detection dogs to locate prion-infected feces in the field (Mallikarjun et al. 2023) may be able to detect the arrival of CWD in a new location earlier than by relying on opportunistic hunter-kill and found carcass testing alone, allowing managers to activate mitigation plans (e.g., culling, selective harvest) and increasing the chance of avoiding an outbreak.”

Katircioglu SJ. Exploring Environmental Dynamics of Chronic Wasting Disease: Soil Matrix Effects on RT-QuIC Detection and Prion Contamination at Scrapes and Food Plots. Doctoral dissertation. 2025.

Mallikarjun A, Swartz, Ben, Kane, Sarah A., Gibison, Michelle, Wilson, Isabella, Collins, Amanda, et al. Canine detection of chronic wasting disease (CWD) in laboratory and field settings. Prion. 2023;17: 16–28. doi:10.1080/19336896.2023.2169519

------------------------------------------------------------------------------------------------------------------

Reviewer #2: Review for PONE-D-25-40590
The authors utilize movement data from mule deer (tested annually) to understand Chronic Wasting Disease transmission risk from environmental sources. They approach this by addressing five competing hypotheses to explore different transmission pathways, with change in CWD-status as the dependent variable:
1) null hypothesis, disease risk is driven by demographic factors only (e.g., age, PRNP genotype),
2) deer density hypothesis (areas more highly used by deer, used KDE),
3) habitat suitability hypothesis, in which risk is related to covariates that affect habitat selection (used RSF),
4) prion persistence hypothesis, risk is driven by environmental covariates that affect the persistence of prions on the landscape,
5) combination hypothesis, in which covariates representing deer density, habitat suitability, and prion persistence are all important.
This study is relevant and warranted, given the spread of CWD and the implications of disease on many economies and properties. The question of environmental transmission has been an important one that governmental, academic, and private entities are interested in addressing, but often lack the data (e.g., change in infection status) to do so. This marks one of the first occasions where such a dataset is available to assess environmental characteristics.
Below we reply to PLOS One reviewer questions (as posted on their website) and we provide specific comments intended to increase clarity of the manuscript.

PLOS One review questions
1. What are the main claims of the paper and how significant are they for the discipline?
The authors identified that the best model in determining risk of CWD infection status was the combination hypothesis. They identified that significant factors in transmission risk included the individual’s genotype, distance to perennial water source during summer, CTI during summer, CTI during winter, distance to cropland during winter, distance to secondary road (year-round). Risk was higher for deer that spent more time near perennial water sources and secondary roads, and farther away from cropland during winter. Disease risk was also higher for deer that spent more time in flatter and wetter areas during winter, but lower for deer in flatter and wetter areas during summer.
2. Are the claims properly placed in the context of the previous literature? Have the authors treated the literature fairly?
Yes, they specifically explored environmental transmission risk, compared their findings with previous work (e.g., percent clay content) and expanded upon previous work by creating risk maps with several environmental factors contributing to infection risk. The authors should consider adding a limitations paragraph in their discussion (i.e., use of only female mule deer).
3. Do the data and analyses fully support the claims? If not, what other evidence is required?
Yes, the data and analyses support the claims.
4. PLOS One encourages authors to publish detailed protocols and algorithms as supporting information online. Do any particular methods used in the manuscript warrant such treatment? If a protocol is already provided, for example for a randomized controlled trial, are there any important deviations from it? If so, have the authors explained adequately why the deviations occurred?
Not applicable.
5. If the paper is considered unsuitable for publication in its present form, does the study itself show sufficient potential that the authors should be encouraged to resubmit a revised version?
It is suitable for publication with some edits.
6. Are original data deposited in appropriate repositories and accession/version numbers provided for genes, proteins, mutants, diseases, etc.?
Information not disclosed. Authors did not include a data sharing agreement.
7. Does the study conform to any relevant guidelines such as CONSORT, MIAME, QUORUM, STROBE, and the Fort Lauderdale agreement?
Not applicable.
8. Are details of the methodology sufficient to allow the experiments to be reproduced?
Not reproducible with the current details provided, however, suggestions to improve clarity and missing details are provided in the line-by-line comments below.
9. Is any software created by the authors freely available?
Not applicable.
10. Is the manuscript well organized and written clearly enough to be accessible to non-specialists?
The methods and results could use some additional formatting and rearrangement to help with clarity.


Major comments:
• Some of the methods section was difficult to follow for an unfamiliar reader. The authors might consider rearranging the methods to have the following sections: (1) Long-term monitoring, where the mule deer monitoring project is described, (2) separate sections to describe the approaches for each hypothesis, (3) model selection across hypotheses, and (4) methods for creation of the disease risk map.
o If authors pursue the aforementioned, reformatting the results in a similar way would help with flow.

Thank you for this suggestion. We have reorganized the Methods according to this suggestion, and we agree that it is easier to follow and keeps the emphasis on the hypotheses related to the risk models.

• Methods. Citations needed for approaches, including moving window analysis, Euclidean distance, and Euclidean distance decay function; and for packages associated with the KDEs, RSFs, and moving window analysis.

We have clarified our reference to “moving window analysis” by changing this text (line 308 in tracked changes document) to: “We investigated habitat use over a range of spatial scales by calculating the mean of habitat variables within different buffer sizes of each GPS point (using the exactextractr package in R, Baston 2023) for continuous value variables (e.g., heat load index).”

We added the following text to clarify our use of distance decay (line 311 in tracked changes document): “The distance decay function describes how the influence of a feature dissipates as distance from the feature increases. In our case, testing distance decay at different scales for proximity variables such as distance to roads tested whether the effect of roads was influential only at close distances, or if influence was still present far from roads.” We also added a citation to (Nekola and White 1999) for the distance decay function.

We added the following to cite the software used in the KDE analysis (line 250 in tracked changes document): “The KDE analysis was performed using ArcGIS Spatial Analysis toolbox (Copyright 2024 Esri Inc., ArcGIS Pro: 3.4.3).”

Baston D. exactextractr: Fast Extraction from Raster Datasets using Polygons. 2023. Available: 10.32614/CRAN.package.exactextractr

Nekola, J. C., & White, P. S. (1999). The distance decay of similarity in biogeography and ecology. Journal of biogeography, 26(4), 867-878.

• Results. An opening paragraph with overall results (e.g., how many mule deer were positive, how many were migratory, how many had the gene target you were looking at in your models) before the model results

We have added a few sentences with this information at the beginning of the Results section (line 539 in tracked changes document), as well as moved Table 1 (now Table 2) to this section.

Minor comments:
Ln 14-16: CWD can also be spread vertically

Now reads “Chronic wasting disease is a prion disease of cervids that is spread to uninfected individuals through direct transmission (contact with infected individuals), vertical transmission (from mother to calf), or indirect transmission (exposure to contaminated environments)”

Ln 30. “Results of risk models also indicated increased risk in areas not associated with high resource selection, suggesting that infection risk was also elevated in areas where environmental properties facilitate prion retention” The “not associated with resource selection” does not provide context as to the features of these areas that may facilitate prion submission. If words permit, it would be useful to name a few.

We have added an example of a property that we found to be important in risk models that was not selected-for by deer, to illustrate our point (line 38 in tracked changes document): “We also found increased risk associated with spatial properties that were not selected-for by deer, such as areas where topography collects moisture, suggesting that prion retention mechanisms also play a role in risk.” We have also edited the abstract for clarity and to remain within the word limit.

Ln 110. There is some information in the introduction that could be moved to the Methods. Specifically, starting at “Hypotheses 2 […]” through Ln 125 (the entire end of this paragraph)– this content feels like it can be moved to the methods. I understand the desire to disclose this information early on, as I also wondered how H2 and H3 differed, but perhaps a brief statement that model differences and descriptions are outlined in the methods is sufficient.

We have moved this information to the Methods section as part of the section reorganization.

Ln 125. A transition into the methods would help with flow.

We have added a final sentence to the introduction to summarize the goal of the study and set up the methods (line 129 in tracked changes document): “Our goal with these analyses was to identify specific environmental properties correlated with increased or decreased probability of deer testing positive for CWD. Though our main motivation was to leverage these correlations to predict patterns of CWD transmission risk on the landscape rather than explain causal relationships driving these patterns, our results can also inform predictions about the mechanisms driving CWD risk in this area that can guide future research.”

Ln 132. What portion of the population migrated to the summer range?
We do not have detailed information on what proportion of the total herd migrate each year, but we can calculate what proportion of the collared animals relevant to this analysis migrated each year. We have added the following text to the Resource Selection Function section of the Methods (line 306 in tracked changes document): “Of the GPS-collared individuals investigated in this study, approximately 45-55% of animals migrated each year.”

Ln 138-139. Can you please provide a reference for deer aging based on tooth wear. Also, what was the decontamination protocol for the jaw spreader (or equivalent)?

We have added the following citation for estimating age based on tooth eruption and wear (line 169 in tracked changes document):

Robinette WL, Jones DA, Rogers G, Gashwiler JS. Notes on Tooth Development and Wear for Rocky Mountain Mule Deer. The Journal of Wildlife Management. 1957;21: 134–153. doi:10.2307/3797579

We have also added the following citation on the cleaning of surgical equipment (line 172 in tracked changes document): “Surgical equipment was cleaned using methods previously published to prevent iatrogenic transmission (Wild et al. 2002)”

Wild MA, Spraker TR, Sigurdson CJ, O’Rourke KI, Miller MW. Preclinical diagnosis of chronic wasting disease in captive mule deer (Odocoileus hemionus) and white-tailed deer (Odocoileus virginianus) using tonsillar biopsy. J Gen Virol. 2002;83: 2629–2634. doi:10.1099/0022-1317-83-10-2629

Ln 147. Only females included in the study – how is this placed in the context of other literature (M v F) exploring environmental influences of spread and risk of infection?
Some previous studies of environmental influences on CWD risk did account for sex in their models (Nobert et al. 2016, Ruiz et al. 2013, Walter et al. 2011). While these studies found that sex was a significant factor in estimating a deer’s overall risk of CWD, we are not aware of any studies that found a significant effect of the interaction between sex and environmental covariates. That is, males and females likely experience different baseline CWD risk, but the relationship between environmental covariates and risk does not differ by sex (or has not been explicitly tested). Unfortunately, we were not able to test for differences by sex in our study due to the limits of the data that was available, but we believe our study is comparable to previous studies that did control for sex in their models. We have also added caveats to the discussion related to the fact that our study was limited to females (see related comment below).

Ln 149-152. Rigorous filtering was implemented for inclusion in the data set. Each animal needed one-year of GPS that had points pre-post CWD positive, collar had to have consistent reads (no gap in data), and at least 6 lymphoid follicles tested.

- What collar brands were used?
- How many GPS points were required pre- and post- CWD test?

Collars were store-onboard global positioning system from Lotek Wireless Inc., Newmarket, Ontario, Canada. We required that there be approximately one year of data before and after the deer’s first positive CWD test, which we defined as at least 300 days with recorded data points, at least 900 points. We have added these details to the sentences indicated (line 159 in tracked changes document).

Ln 156. Reference to Table 01 should be in the methods

We have moved table 1 to the first paragraph of the Results, since we felt that it fit best with the added paragraph suggested by the reviewer, describing how many deer were in the analysis, how many of each genotype etc. This is now Table 2 after reorganizing sections.

Ln 164- 165. The authors state “expected to attract or deter use by mule deer”, could you please provide citations?

We have added citations for this section, indicating prior studies that have found that deer are attracted or repelled by different habitat characteristics (line 293 in tracked changes document). 

Ln 168. This is the first use of “RSF” but it has not yet been spelled out with the acronym.
We have added the meaning of the acronym here (line 284 in tracked changes document).

Ln 201. Classifying migratory status as behavior is a bit confusing, can you use different terminology here? You use “migratory groups” in the Discussion (Ln 439-440), this might be a better description.

We have changed this and other instances to refer to “migratory groups” instead of behavior.

Ln 217-219. GLMs are defined earlier so no need to write out here. Should also bump the R package citation to earlier.

We have made this change.

Ln 286. It might be helpful to reiterate that behavior types corresponds to migratory groups.
We have made this change.

Discussion. Were any discrepancies observed between your results and previously published literature (e.g., soil clay content) possibly influenced by the limitations associated with the individual animals – specifically, do you think these results may be limited since only females are used? Could this explain some differences observed here relative to past work (are they directly comparable)? Please add a few sentences discussing the limitations of using only females.
While only using females in the model certainly removed a significant portion of the herd from consideration, we don’t think it’s likely that excluding males could explain the discrepancies we saw between our study and previous studies. It is more likely that discrepancies arose from the fact that the previous studies we cited used an individual’s presumed home range to infer the area where infection occurred, while we used specific GPS points. Both approaches are imperfect methods for estimating exposure risk and where the deer was when infection occurred, and it is possible that we found different results because we made different assumptions and used different methods. However, we agree that our results should only apply to females since only female data was used, and it is established in the literature that males usually have higher CWD prevalence than females, suggesting different pathways of infection. It is hypothesized that this is more likely due to differences in social behavior, particularly during rut, and would therefore be the result of direct transmission rather than indirect transmission from environmental contaminants. We predict that if we did have the ability to model males in the same way we modeled females, we would find weaker relationships between habitat use and probability of infection. We have added a paragraph to the discussion that clarifies that we were not able to disentangle direct from indirect transmission in this study, and that our results apply specifically to females (line 805 in tracked changes document).

Table 1. Additional information is needed in this table caption for it to be stand-alone table (also may fit better in the result section)

The caption now reads (line 544 in tracked changes document): “Numbers of GPS-collared mule deer used in models predicting relative risk of a deer newly testing positive for chronic wasting disease based on habitat visited by deer. Deer individuals were identified by genotype and were either homozygous for serine at codon 225 (“SS” genotype), or were heterozygous/homozygous for phenylalanine at codon 225 (“*F” genotype). All individuals included in models did not test positive for CWD on first capture. The total number of individuals in each genotype group is further broken down into individuals that never tested positive for chronic wasting disease for the duration of the study (“Remained CWD-“) and those that had tested positive by the end of the study (“Became CWD+”). All individuals in this analysis are female.”

Table 2. Are the column labels supposed to reflect the hypotheses? The language used in-text earlier in the manuscript made it seem like RSF models were used to test habitat suitability hypothesis (Ln 119-120), so what are the columns representing (which model?) It is assumed that the “risk model: prion” is to address hypothesis 4 (prion persistence)?
Yes, the column titled “risk models: habitat suitability” represents variables used to address hypothesis 3 and the column titled “risk models: prion persistence” represents variables used to address hypothesis 4. We have simplified this table by removing the column that lists the variables that were included in the RSF analysis, as this analysis was not the main focus of the paper, and we instead list the specific variables that were used in the RSF analysis in the supplemental material. We have also added more information to the caption to clarify that the two columns represent two of the hypotheses (line 350 in tracked changes document).

We are grateful for the opportunity to review this manuscript and applaud the authors on their excellent work.
Alynn Martin & Ashlyn Halseth-Ellis

[NOTE: If reviewer comments were submitted as an attachment file, they will be attached to this email and accessible via the submission site. Please log into your account, locate the manuscript record, and check for the action link "View Attachments". If this link does not appear, there are no attachment files.]

While revising your submission, please upload your figure files to the Preflight Analysis and Conversion Engine (PACE) digital diagnostic tool, <https://pacev2.apexcovantage.com/>. PACE helps ensure that figures meet PLOS requirements. To use PACE, you must first register as a user. Registration is free. Then, login and navigate to the UPLOAD tab, where you will find detailed instructions on how to use the tool. If you encounter any issues or have any questions when using PACE, please email PLOS at [figures@plos.org](mailto:figures@plos.org). Please note that Supporting Information files do not need this step.
